# Carbon dioxide regulates cholesterol levels through SREBP2

**DOI:** 10.1371/journal.pbio.3002367

**Published:** 2023-11-15

**Authors:** Nityanand Bolshette, Saar Ezagouri, Vaishnavi Dandavate, Iuliia Karavaeva, Marina Golik, Hu Wang, Peter J. Espenshade, Timothy F. Osborne, Xianlin Han, Gad Asher

**Affiliations:** 1 Department of Biomolecular Sciences, Weizmann Institute of Science, Rehovot, Israel; 2 The Sam & Ann Barshop Institute for Longevity & Aging Studies, University of Texas Health Science Center, San Antonio, Texas, United States of America; 3 Department of Cell Biology, Johns Hopkins University School of Medicine, Baltimore, Maryland, United States of America; 4 Institute for Fundamental Biomedical Research, Johns Hopkins All Children’s Hospital, and Medicine in the Division of Endocrinology, Diabetes and Metabolism of the Johns Hopkins University School of Medicine, Petersburg, Florida, United States of America; Ecole polytechnique federale de Lausanne Faculte des sciences de la vie, SWITZERLAND

## Abstract

In mammals, O_2_ and CO_2_ levels are tightly regulated and are altered under various pathological conditions. While the molecular mechanisms that participate in O_2_ sensing are well characterized, little is known regarding the signaling pathways that participate in CO_2_ signaling and adaptation. Here, we show that CO_2_ levels control a distinct cellular transcriptional response that differs from mere pH changes. Unexpectedly, we discovered that CO_2_ regulates the expression of cholesterogenic genes in a SREBP2-dependent manner and modulates cellular cholesterol accumulation. Molecular dissection of the underlying mechanism suggests that CO_2_ triggers SREBP2 activation through changes in endoplasmic reticulum (ER) membrane cholesterol levels. Collectively, we propose that SREBP2 participates in CO_2_ signaling and that cellular cholesterol levels can be modulated by CO_2_ through SREBP2.

## Abstract 2

In mammals, oxygen and carbon dioxide levels are tightly regulated and are altered under various pathological conditions, but little is known regarding the pathways involved in CO2 signaling and adaptation. This study provides new insight into the interaction between carbon dioxide, SREBP2 activation and regulation of cholesterol levels, suggesting that cellular cholesterol levels can be modulated by carbon dioxide through SREBP2.

## Introduction

A fundamental process in mammalian physiology is oxygen (O_2_) uptake from the environment into cells in exchange of carbon dioxide (CO_2_), a byproduct of energy generation upon aerobic respiration. Oxygen is an essential substrate for cellular metabolism and bioenergetics and is indispensable for normal physiology and survival. Consequently, mammals have developed mechanisms to sense O_2_ levels and regulate O_2_ consumption in order to cope with conditions of insufficient O_2_ supply. A principal regulator in the response to low oxygen levels is the hypoxia-inducible factor (HIF), which participates in sensing of low oxygen levels and subsequently activates a transcriptional program that facilitates cellular adaptation to changes in oxygen levels [1–3]. While the cellular response to oxygen levels is well characterized, relatively little is known regarding the mechanisms that participate in response to changes in CO_2_ levels. It is noteworthy that CO_2_ plays various critical roles in mammalian physiology including regulation of blood pH, respiratory drive, and O_2_ affinity for hemoglobin [4]. Under physiological conditions, arterial blood CO_2_ levels are tightly maintained approximately 35 to 45 mm Hg (approximately 5%). Altered CO_2_ levels are associated with the pathophysiology of various diseases such as chronic obstructive pulmonary disease (COPD) and obstructive sleep apnea (OSA) as well as impaired wound healing and fibrosis [5–7].

Carbon dioxide molecules are transported in the blood from body tissues to the lungs by one of 3 methods: dissolution directly into the blood, binding to hemoglobin, or carried as a bicarbonate ion. About 10% of CO_2_ is dissolved in the plasma, a small fraction is bound to hemoglobin, while the majority (about 85%) is carried as a part of the bicarbonate buffer system [4]. In aqueous solution, CO_2_ reacts with the water to form carbonic acid (H_2_CO_3_), which is readily buffered by the bicarbonate buffer system to maintain the pH levels within the physiological range [8].

To identify signaling pathways that regulate gene expression in response to changes in CO_2_ levels, and hence participate in CO_2_ sensing, we employed a cell culture setup alongside high-throughput transcriptomic and biochemical analyses. We found that CO_2_ activates a distinct transcriptional response that is dependent on SREBP2, a key regulator of cholesterol biosynthesis, to regulate the expression of cholesterogenic genes and cholesterol accumulation. SREBP2 regulation by CO_2_ is likely mediated by changes in endoplasmic reticulum (ER) membrane cholesterol levels. We, thus, propose that SREBP2 plays a role in cellular CO_2_ signaling and that SREBP2 regulation of cholesterol levels can be modulated by changes in CO_2_ levels.

## Results

### The transcriptional response to low CO_2_ differs from pH

**Fig 1:**
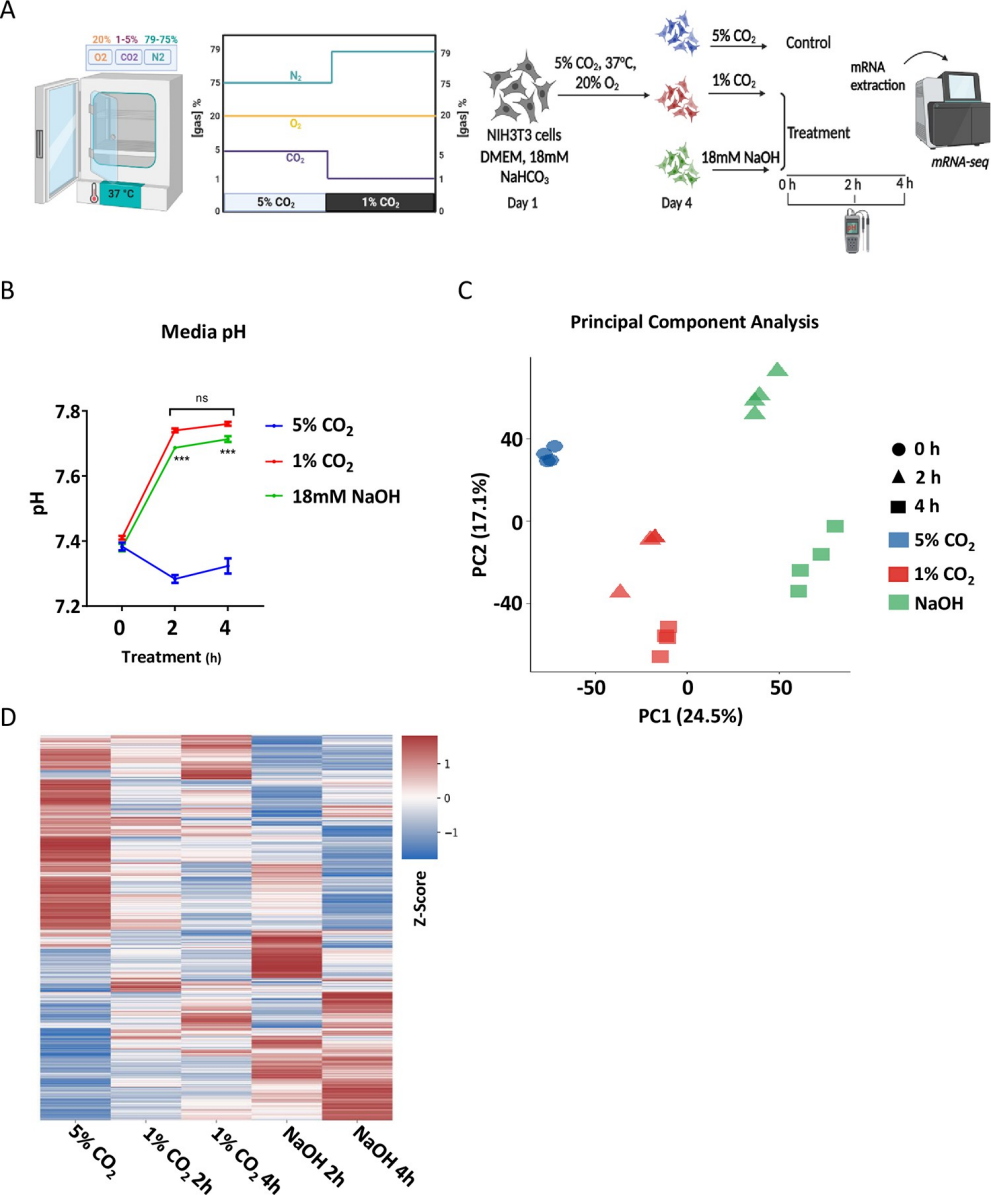
The transcriptional response to low CO_2_ levels compared to NaOH treatment. (**A**) A schematic depiction of the experimental design. NIH3T3 cells were cultured in media B (as detailed in method) at 37°C with 5% CO_2_ and 20% oxygen. On day 4, cells were either untreated or treated with 18 mM NaOH or shifted to a special incubator (Coy Labs, USA) with 1% CO_2_ and 20% oxygen. Cells and media were collected 2 and 4 h post treatment. RNA was extracted and analyzed by RNA sequencing (*n* = 4 for each time point per condition). (**B**) pH measurements of the growth media (mean ± SE, *n* = 3 biological replicates for each time point per condition, ***P < 0.001, nonsignificant (ns), two-sided Student’s *t* test). (**C**) PCA. (**D**) Heatmap representation of genes that were significantly altered (see Methods) between time points within conditions. Data are presented as row z-scores of the expression per condition. See also S1 Fig and S1 Table. The data underlying the graphs shown in the figure is included in S1 Data. Graphical illustrations were generated with BioRender.com. PCA, principal component analysis.

To identify signaling pathways that participate specifically in CO_2_ sensing and not changes in pH, we examined the global transcriptional response of cultured cells to reduction in CO_2_ levels from 5% to 1%. We used special chambers with CO_2_, O_2_ and temperature controls [9]. Temperature, O_2_ and CO_2_, levels were continuously monitored throughout the experiment with constant temperature of 37°C and 20% O_2_. While CO_2_ levels were modulated by replacing them with the inert nitrogen gas and were kept either at 5% or 1% (Fig 1A).

As aforementioned, once CO_2_ reacts with aqueous solution it forms carbonic acid and acidifies it. Since the reduction in CO_2_ levels from 5% to 1% resulted in alkaline condition, we also used 18 mM NaOH to alkalize the media as a control for changes that are purely pH-dependent. Importantly, under both conditions, namely 1% CO_2_ or 18 mM NaOH, the media pH at 2 and 4 h post exposure was similar (approximately 7.7) and differed from that of control cells (5% CO_2_), which maintained pH across the normal physiological range (approximately 7.3) (Fig 1B). NIH3T3 cells (a fibroblast cell line that was isolated from mouse NIH/Swiss embryos) were harvested 2 and 4 h post exposure, RNA was extracted and analyzed by RNA-sequencing. The transcriptomic analysis revealed that the transcriptional response differed between the low CO_2_ exposure and the alkaline conditions, even though the pH was similar (Figs 1C and 1D and S1). Notably, principal component analysis (PCA) and unsupervised clustering analyses (Fig 1C and 1D) clearly discriminated between exposure to low CO_2_ versus NaOH treatment. NaOH treatment induced a prominent effect on gene expression with 2,697 genes showing differential response (P adj. < = 0.05, |log2FC| > = 1, baseMean > = 5), with 1,320 up- and 1,377 down-regulated. While exposure to a low CO_2_ level led to a milder effect on gene expression (1,328 genes with 685 up- and 643 down-regulated) (S1A and S1B Fig). Although, both the up- and down-regulated genes overlapped between the CO_2_ and NaOH groups, we found in line with the PCA and cluster analyses that a significant number of genes are uniquely altered in response to CO_2_ (S1B Fig).

Overall, our analyses show that under similar alkaline pH, the transcriptional response differs between low CO_2_ and NaOH treatments. Thus, supporting a distinct mechanism that is activated in response to changes in CO_2_ levels to regulate gene expression.

### CO_2_ alters the expression of genes that participate in cholesterol biosynthesis

**Fig 2:**
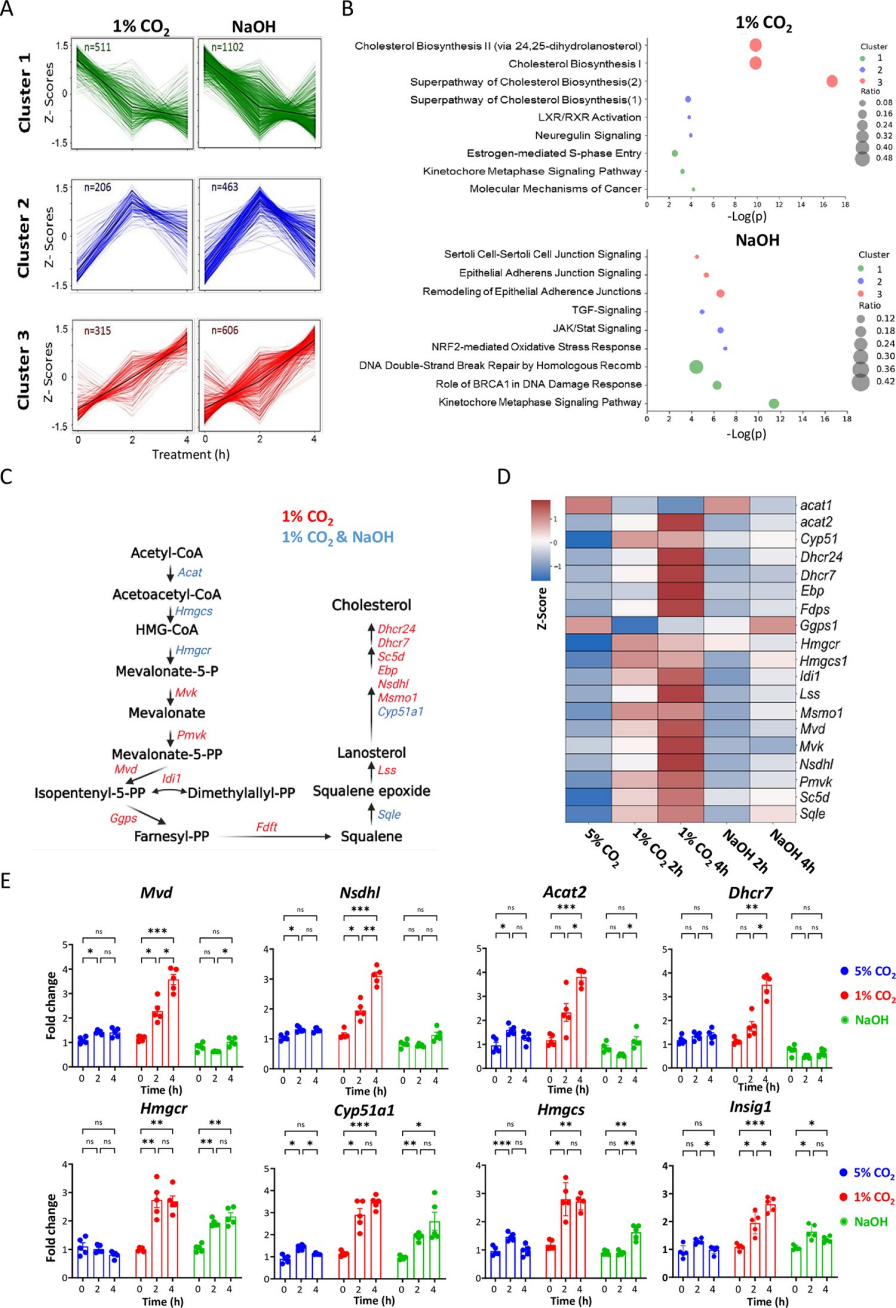
Low CO_2_ levels specifically induce the expression of genes related to cholesterol biosynthesis. **(A)** K-means unsupervised clustering of significant genes for each of the conditions. Black line represents the mean z-score (for gene lists see S2 Table). **(B)** Pathway enrichment analysis was performed using the IPA tool for the genes included in each of the clusters for 1% CO_2_ or 18 mM NaOH treatments. Presented are the top 3 enriched pathways in each cluster based on *P* value (for full list of pathways, see S3 Table). **(C)** Schematic illustration of the cholesterol biosynthesis pathway alongside genes that were significantly affected by the treatments. Color indicates on the condition in which the genes are affected. **(D)** Heatmap representation of cholesterogenic genes that were significantly affected by any of the conditions presented. Data are presented as row z-score of the average expression per condition (*n* = 4 biological replicates). **(E)** Quantitative PCR analysis of cholesterogenic gene expression levels from NIH3T3 cells treated with 1% CO_2_ or 18 mM NaOH (mean ± SE, *n* = 5 biological replicates per time point per condition, *P < 0.05, **P < 0.01, ***P < 0.001, nonsignificant (ns), two-way ANOVA with Tukey’s post hoc test) (see also S2 and S3 Figs and S2 and S3 Tables). The data underlying the graphs shown in the figure is included in S1 Data. IPA, ingenuity pathway analysis.

To identify potential transcription factors that participate in gene expression regulation in response to CO_2_ or NaOH, we took an advantage of our time course analysis and performed unbiased cluster analysis (Fig 2A). We identified 3 major clusters; *Cluster 1*: Transcripts that were monotonically down-regulated (CO_2_ or NaOH; 511 and 1,102, respectively); *Cluster 2*: Transcripts that were up-regulated exclusively after 2 h (CO_2_ or NaOH; 206 and 463, respectively); and *Cluster 3*: Transcripts that were monotonically up-regulated (CO_2_ or NaOH; 315, and 606, respectively). Next, to uncover related biological processes affected by each treatment, we performed pathway enrichment analysis on each cluster. Remarkably, we found that cholesterol biosynthesis and its related processes are highly enriched in response to CO_2_ but not to NaOH, specifically in cluster 3 which includes the monotonically up-regulated transcripts (Fig 2B). These findings indicated that low CO_2_ induces the expression of genes implicated in cholesterol metabolism and that this effect is not a mere response to alkaline conditions, as it was not apparent upon NaOH treatment. This prompted us to specifically examine expression pattern of enzymes involved in de novo cholesterol biosynthesis based on our RNA-sequencing data. The vast majority of enzymes involved in different stages of cholesterol biosynthesis were up-regulated in cells exposed to low CO_2_. Notably, the induction of these transcripts was mostly absent in NaOH-treated cells (Fig 2C and 2D). Furthermore, analysis of cholesterogenic gene expression by qPCR showed that in most cases their transcript levels are specifically induced by low CO_2_ levels but not upon NaOH treatment (Fig 2E). These results were in line with the above detailed RNA-sequencing analysis. A similar trend was observed in hepatocyte murine cell line (Hepa1c1) (S2A Fig). In addition, these effects were recapitulated in primary tail fibroblasts and primary muscles, but not in primary white or brown adipocytes (S2A Fig).

Next, we examined the effect of hypercapnia, namely elevated CO_2_ level, on cholesterogenic gene expression. Cells were exposed to increased CO_2_ level (i.e., 10%) for 2 and 4 h and the transcript levels of cholesterogenic genes were analyzed by qPCR. Here again, O_2_ level was maintained constant at 20% using our CO_2_, O_2_ and temperature-controlled chambers. High CO_2_ levels elicited the opposite effect to lower CO_2_ levels and the expression levels of cholesterogenic genes were suppressed (S2B Fig). Comparison of gene expression data of THP-1 monocytes exposed to 10% CO_2_ [10] with our NIH3T3 cells data (1% CO_2_ exposure) showed a small overlap in the responsive genes (S3A Fig). Yet, this small group included cholesterogenic genes (e.g., *Ldlr*, *Idi1*, *Insig1*, *Hmgcs1*, *Dhcr7*) and their response was in line with our findings, namely 10% CO_2_ repressed of their expression (e.g., *Insig1*, *Hmgcs1*) (S3B Fig).

Taken together, our analyses reveal that alteration of CO_2_ levels from the physiological range modulate the expression of genes involved in cholesterol homeostasis. Reduced and elevated CO_2_ levels activate and repress their expression, respectively.

### SREBP2 is activated in response to low CO_2_ to induce the expression of cholesterogenic genes

**Fig 3:**
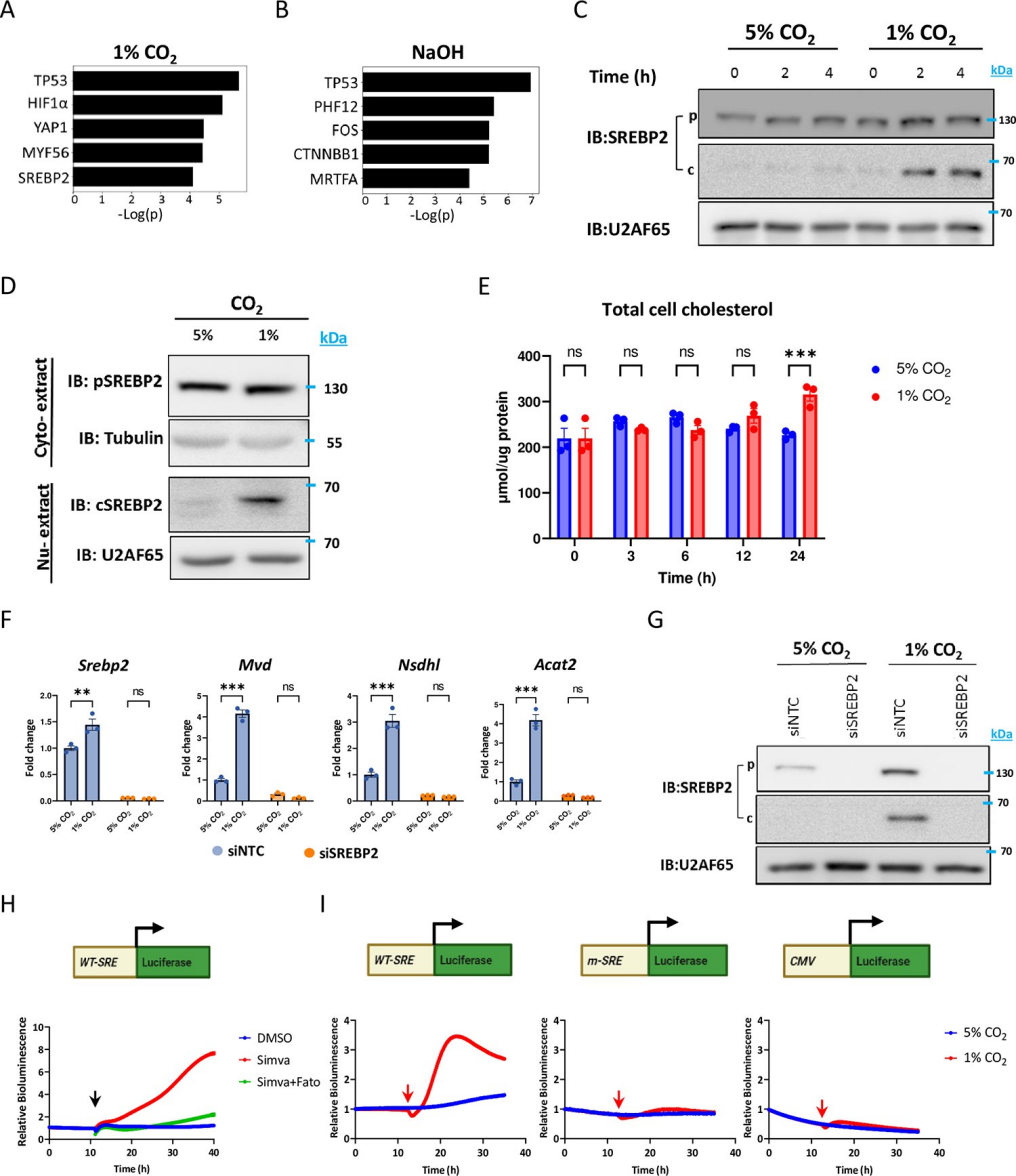
Low CO_2_ levels activate SREBP2 and induce the expression of cholesterogenic genes through SRE. **(A, B)** Upstream regulator analysis was performed with IPA for clusters 2 and 3 within each condition. The top transcription factors, with the highest *p*-value, are presented (for full list, see S4 Table). **(C)** Immunoblot of total cell extracts from NIH3T3 cells exposed to either 5% CO_2_ or 1% CO_2_ p—SREBP2 precursor (approximately 126 kD); c—SREBP2 cleaved form (approximately 68 kD) (pooled sample of *n* = 3 biological replicates). **(D)** Immunoblot of cytoplasmic (Cyto-extract) and nuclear fractions (Nu-extract) from NIH3T3 cells exposed to either 5% or 1% CO_2_ for 4 h (pooled sample of *n* = 3 biological replicates). **(E)** Total cholesterol quantification of NIH3T3 cells that were exposed to 5% or 1% CO_2_ for 0, 3, 6, 12, 24 h (mean ± SE, *n* = 3 biological replicates per condition, ***P < 0.001, nonsignificant (ns), two-way ANOVA with Bonferroni’s multiple comparisons test). **(F)** Quantitative PCR analysis for expression levels of cholesterol biosynthesis-related genes from control (siNTC) or SREBP2 silenced (siSREBP2) NIH3T3 cells exposed to 5% or 1% CO_2_ for 4 h (mean ± SE, *n* = 3 biological replicates per condition, ***P < 0.001, **P < 0.01, nonsignificant (ns), two-way ANOVA with Bonferroni’s multiple comparisons test). **(G)** Immunoblot of NIH3T3 cells under the same condition as in **(F)** (pooled sample of *n* = 3 biological replicates). **(H)** Bioluminescence recordings from NIH3T3 cells transfected with SRE-Luc reporter plasmid (WT-SRE) and exposed to DMSO (control), 20 μm simvastatin or 20 μm simvastatin + 20 μm fatostatin, black arrow indicates the time of treatment (mean ± SE, *n* = 3 biological replicates per condition, AUC for control 1.14 ± 0.009, simvastatin 4.05 ± 0.06 (*P* < 0.0001), simvastatin + fatostatin 1.33 ± 0.04 (*P* < 0.0001), two-sided Student’s *t* test). **(I)** Bioluminescence recordings from NIH3T3 cells transfected with WT SRE-Luc, mutant SRE-Luc, or control vector (CMV-Luc), and exposed to either 5% or 1% CO_2_, the red arrow indicates the shift in CO_2_ levels (mean ± SE, *n* = 6 biological replicates per condition, AUC for SRE Luc 5% CO_2_ 1.24 ± 0.03, 1% CO_2_ 2.77 ± 0.01 (*P* < 0.0001), mSRE Luc 5% CO_2_ 0.83 ± 0.02, 1% CO_2_ 0.91 ± 0.01 (*P* < 0.002), CMV Luc 5% CO_2_ 0.33 ± 0.007, 1% CO2 0.42 ± 0.01 (*P* < 0.001), two-sided Student’s *t* test) (see also S4 and S5 Figs). The data underlying the graphs shown in the figure is included in S1 Data. AUC, area under curve; IPA, ingenuity pathway analysis; SRE, sterol regulatory element.

SREBP2 is a key transcriptional regulator of genes involved in cholesterol biosynthesis [11,12]. In response to changes in cholesterol levels, SREBP2 translocates from the ER to the Golgi, where subsequent cleavage occurs and the N-terminal form of SREBP2 shuttles to the nucleus and activates the expression of transcripts involved in cholesterol biosynthesis [13]. Our transcription factor analysis predicted SREBP2 among the top potential transcriptional regulators for the expression of genes that are up-regulated (clusters 2 and 3) upon exposure to low CO_2_ but not in response to NaOH treatment (Fig 3A and 3B).

We, therefore, hypothesized that SREBP2 is activated in response to low CO_2_ to induce the expression of enzymes involved in cholesterol biosynthesis. To test this, cultured cells were exposed to low CO_2_ and SREBP2 was analyzed by SDS-PAGE and immunoblot analysis. We found that the cleaved form of SREBP2 (approximately 68 kD) accumulates 2 and 4 h following exposure to low CO_2_ levels (Fig 3C). This effect was specific to low CO_2_ and not to alkalic pH as it was not observed in NaOH-treated cells (S4A Fig). Biochemical nuclear-cytoplasmic fractionation further showed that the cleaved form of SREBP2 accumulates in the nucleus upon exposure to low CO_2_ levels (Fig 3D). Together, our findings indicate that the SREBP2 signaling pathway is activated upon exposure to low CO_2_ levels. To examine the functional consequence of SREBP2 and its downstream gene activation, we performed a time course analysis (0, 3, 6, 12, and 24 h) and measured cholesterol levels in cells cultured either at 5% or 1% CO_2_. Upon 24 h exposure to low CO_2_ levels, cells accumulated cholesterol, in line with SREBP2 activation and elevated the expression of cholesterogenic genes (Fig 3E). Next, we asked whether the induction of cholesterogenic genes under low CO_2_ is SREBP2-dependent. To this end, cells were transfected with either control siRNA (siNTC-Non Template Control) or siRNA against mouse SREBP2 (siSREBP2) and were exposed either to 1% CO_2_ or 5% CO_2_ for 4 h. As expected, SREBP2 was undetectable in siSREBP2-silenced cells under both 5% and 1% CO_2_ and the basal expression levels of SREBP2 target genes was lower (Fig 3F and 3G). Control cells showed accumulation of the cleaved form of SREBP2 upon 1% CO_2_ as well as induction of its cholesterogenic target genes (Fig 3F and 3G). Importantly, the induction of cholesterogenic genes was completely abolished in SREBP2 silenced cells under low CO_2_ levels, indicating that the effect is SREBP2-dependent (Fig 3F). We also identified several transcripts that are induced upon low CO_2_ levels in our gene expression analysis yet their induction was SREBP2-independent (S4B Fig). It is conceivable that the response to low CO_2_ levels is coordinated through the concerted action of several transcription regulators and is not exclusively SREBP2-dependent. Overall, our results suggest that low CO_2_ levels elicit SREBP2 cleavage and nuclear accumulation to induce the expression of its target genes, primarily cholesterogenic genes and consequently cholesterol accumulation.

### Low CO_2_ activates gene expression through a sterol regulatory element

SREBP2 activates the transcription of its downstream targets by binding to a specific region on the promoter sequence known as sterol regulatory element (SRE) [14]. To examine whether low CO_2_ levels can activate gene expression through an SRE, we employed an SRE reporter assay. This reporter is based on the HMG-CoA synthase promoter sequence harboring SRE that drive the expression of a firefly luciferase [15]. Cells were transfected with the SRE reporter and bioluminescence was continuously monitored. Consistent with the activation of SRE by SREBP2, treatment with simvastatin, which inhibits de novo cholesterol biosynthesis [16] and activates SREBP2, resulted in increased bioluminescence. This effect was suppressed upon co-administration of fatostatin (Fig 3H), which inhibits SREBP2 ER-to-Golgi translocation [17].

Then, we tested the effect of low CO_2_ on the reporter activity. In line with above-described findings, a decrease in CO_2_ levels from 5% to 1% induced an increase in bioluminescence of cells expressing the wild-type reporter (pSynSRE-T-Luc) (Fig 3I). A decrease in CO_2_ levels had no effect on the bioluminescence of cells expressing either a mutant reporter (pSynSRE-Mut-T-Luc) [18] or a control luciferase reporter (pcDNA3-Luc) (Fig 3I). Consistently, an increase in CO_2_ levels from 5% to 10% markedly suppressed the bioluminescence from cells expressing a wild type but not a control luciferase reporter (S5A Fig).

In our bioluminescence reporter assays, we observed an initial minor response that was not SRE-specific and was evident in the control reporters as well. This unspecific response likely stems from the effect of pH changes on bioluminescence in general [19].

Next, we employed our reporter assay to examine whether SRE activation by low CO_2_ is reversible. To this end, cells expressing wild-type SRE reporter were exposed to either constant 5% as a control or interchanging 5% to 1% CO_2_ levels and bioluminescence was continuously recorded. A shift in CO_2_ levels from 5% to 1% increased the bioluminescence levels. This increase was reduced back to basal levels once CO_2_ levels were shifted to 5% (S5B Fig). This result indicates that CO_2_ reversibly modulate SRE activation and likely SREBP2 activation.

Taken together, our results suggest that an intact SRE is sufficient for the transcriptional response to changes in CO_2_ levels and the effects of CO_2_ levels on it are reversible.

### Stability of the mature cleaved form of SREBP2 is not affected by low CO_2_ levels

SREBP2 translocates from the ER-to-Golgi and subsequently reaches the nucleus to induce gene expression. The exit of SREBP2 from the ER is regulated by sterol levels via SREBP cleavage-activating protein (SCAP) and insulin-induced gene (INSIG). Low ER cholesterol levels destabilize INSIG-SCAP interaction and successively enable the SREBP2-SCAP complex to translocate from the ER to Golgi where SREBP2 is cleaved [20]. The mature N-terminal cleaved form of SREBP2 then shuttles to the nucleus [13] to activate gene expression as aforementioned through SRE sites on target genes [21].

Hitherto, we showed that upon low CO_2_ levels SREBP2 is cleaved, the N-terminal cleaved form accumulates in the nucleus and can activate gene expression though an intact SRE site (Fig 3). To identify the signaling node though which SREBP2 is activated in response to low CO_2_ levels, we systematically examined the different steps in the SREBP2 signaling pathway (S6A Fig) comparing sterol depletion with exposure to low CO_2_ levels.

In the nucleus, the levels of mature cleaved form of SREBP2 are regulated by its proteasomal degradation [22] as stabilization of the nuclear form by proteasome inhibition or defective polyubiquitination actively induce its target genes [23,24]. We hypothesized that low CO_2_ levels might alter nuclear SREBP2 turnover and thereby induce its nuclear accumulation and target gene expression. To test this, we exogenously expressed in cultured NIH3T3 cells a FLAG-tagged truncated mature SREBP2 fragment (FLAG N-SREBP2) [25], which was shown to localize in the nucleus [26]. Cells were exposed either to sterol depletion upon methyl-β-cyclodextrin (MBCD) treatment or to 1% CO_2_ levels. Total protein extracts were prepared and analyzed by immunoblot with either anti-SREBP2 or anti-FLAG antibody to detect the endogenous or the exogenously expressed truncated forms, respectively. Both MBCD treatment and exposure to low CO_2_ induced the accumulation of the endogenous cleaved form of SREBP2 (S6B Fig). However, neither treatment affected the levels of the exogenously expressed cleaved form (i.e., FLAG N-SREBP2) (S6C Fig), suggesting that low CO_2_ levels, similar to sterol depletion by MBCD do not affect the nuclear stability of the cleaved mature form of SREBP2.

### Low CO_2_ levels induce the ER-to-Golgi translocation of SREBP2

SCAP-SREBP2 ER-to-Golgi translocation is a critical step in SREBP2 activation and subsequent induction of its target genes. To examine whether the activation of SREBP2 upon low CO_2_ is dependent on its ER-to-Golgi trafficking, we employed fatostatin, which pharmacologically blocks the ER-to-Golgi transport of SCAP-SREBP2 [17]. Cells were exposed to either fatostatin or DMSO as control under 5% or 1% CO_2_. Low CO_2_ levels induced the accumulation of the mature cleaved form of SREBP2. Importantly, this effect was blocked in the presence of fatostatin (S6D Fig). Consistently, the induction of SREBP2 target genes in response to low CO_2_ levels was eliminated in the presence of fatostatin (S6E Fig). This result indicated that ER-to-Golgi trafficking is necessary for activation of SREBP2 by low CO_2_ levels.

As aforementioned, SCAP regulates SREBP2 transport in a sterol-dependent fashion as it retains the SCAP-INSIG-SREBP2 complex in the ER membrane and inhibits the subsequent processing of SREBP2, namely, its cleavage and ER-Golgi translocation [27]. We, therefore, examined whether activation of SREBP2 by low CO_2_ levels is also SCAP-sensitive. We employed siRNA to knockdown SCAP and exposed control (siNTC) or SCAP knockdown (siSCAP) cells to low CO_2_ levels (i.e., 1%). Low SCAP levels in cultured cells were shown to suppress SREBP2 proteolysis and expression of SREBP2 downstream target genes [28,29]. The induction of SREBP2 target genes in response to low CO_2_ levels was as well suppressed in SCAP-deficient cells likely due to inhibition of SREBP2-SCAP ER-to-Golgi translocation (S6F Fig).

Together, our analyses suggest that activation and induction of SREBP2 target genes upon low CO_2_ levels is dependent on ER-to-Golgi trafficking and regulated by SCAP. Hence, it seems to follow the canonical pathway of SREBP2 activation as in response to low sterol levels.

### Low CO_2_ levels reduces ER cholesterol levels

The main driver of SREBP2 signaling pathway is reduction in ER cholesterol levels. Hitherto, activation of SREBP2 by low CO_2_ levels followed similar steps in the canonical SREBP2 pathway as upon sterol depletion. These findings raised the following questions: (i) Does SREBP2 activation by low CO_2_ levels depend on cellular cholesterol levels; and (ii) do CO_2_ levels affect cellular cholesterol levels?

**Fig 4:**
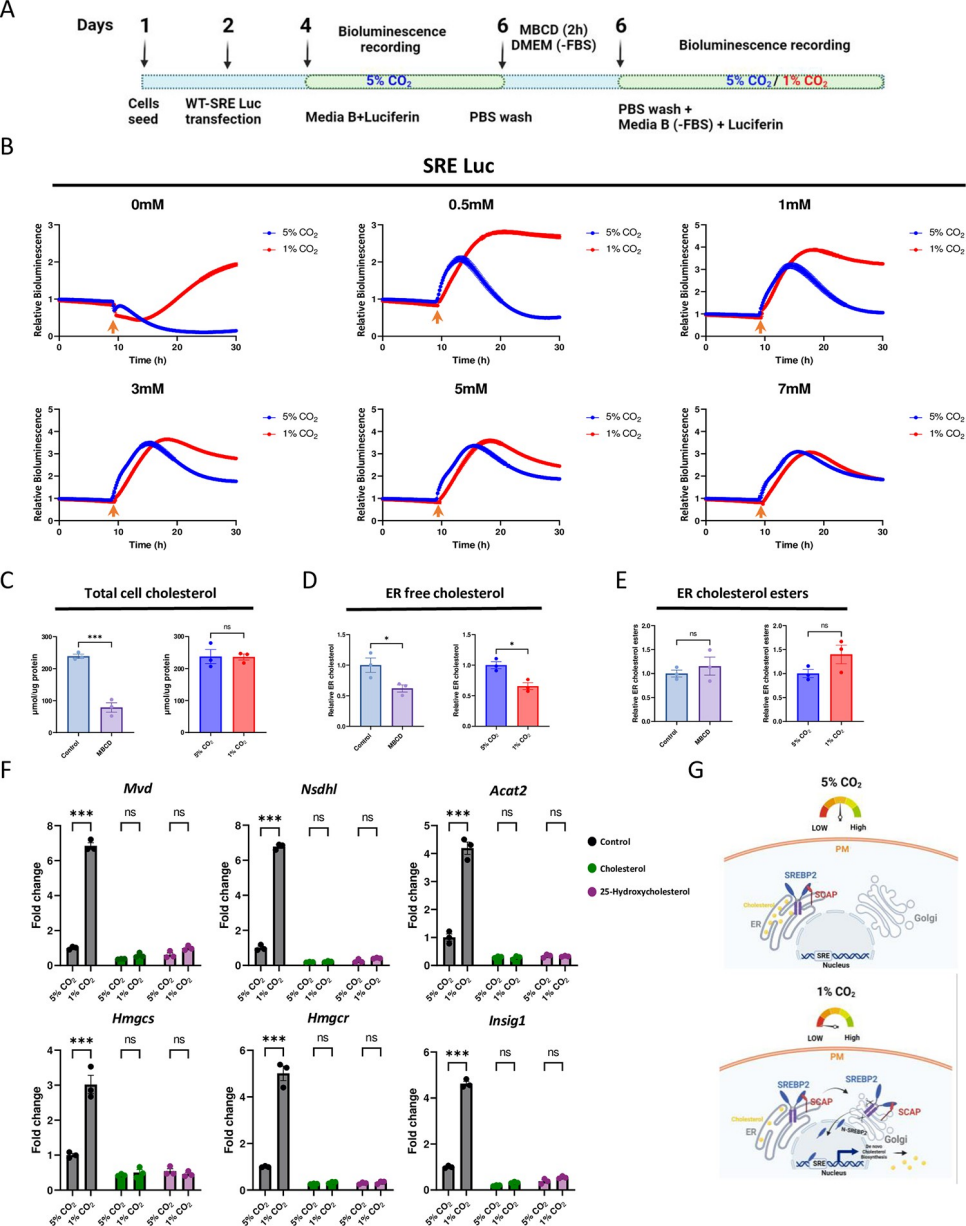
Exposure to low CO_2_ levels decreases ER cholesterol levels. **(A)** Schematic depiction of the experimental design. NIH3T3 cells transfected with reporter plasmid were treated with different MBCD concentrations for 2 h, followed by exposure to either 5% or 1% CO_2_, and bioluminescence levels were continuously recorded in a medium B (without serum) containing luciferin. **(B)** Bioluminescence recordings from the different conditions as depicted in **(A)**, the arrow indicates the time CO_2_ was shifted from 5% to 1% (mean ± SE, *n* = 4 biological replicates per condition, AUC for 0 mM 0.34 ± 0.007, 1.59 ± 0.004 (*P* < 0.0001), 0.5 mM 1.00 ± 0.023, 2.68 ± 0.04 (*P* < 0.0001), 1 mM 1.66 ± 0.07, 3.34 ± 0.04 (*P* < 0.0001), 3 mM 2.06 ± 0.02, 2.79 ± 0.07 (*P* < 0.0001), 5 mM 2.02 ± 0.05, 2.43 ± 0.06 (*P* < 0.002), 7 mM 1.85 ± 0.01, 1.77 ± 0.02 (*P* < 0.02) two-sided Student’s *t* test). **(C)** Total cholesterol quantification (with fluorometric assay kit) in NIH3T3 cells depleted with sterols for 2 h or exposed to different CO_2_ levels for 4 h (mean ± SE, *n* = 3 biological replicates per condition, **P < 0.01, nonsignificant (ns), two-sided Student’s *t* test). **(D, E)** The free cholesterol and cholesterol ester levels in the ER membrane from the cells as in **(C)**, were quantified with shotgun lipidomics analysis (see S7 Fig and S5 Table) (mean ± SE, *n* = 3 independent experiments, **P < 0.01, *P < 0.05, nonsignificant (ns) two-sided Student’s *t* test). **(F)** qPCR analysis for cholesterogenic genes from NIH3T3 cells exposed to cholesterol (50 μm) or 25-hydroxycholesterol (10 μm) under 5% or 1% CO_2_ for 4 h (mean ± SE, *n* = 3 biological replicates per condition, ***P < 0.001, nonsignificant (ns), two-way ANOVA with Bonferroni’s multiple comparisons test). **(G)** A schematic model; in cells under normal physiological CO_2_ levels (5%) ER cholesterol levels are unaffected and SREBP2 is retained in the ER membrane. However, under low CO_2_ levels (1%), ER cholesterol levels are decreased, inducing SREBP2 activation and subsequent activation of cholesterol biosynthesis related genes through SRE region on their gene promoter. The data underlying the graphs shown in the figure is included in S1 Data. Graphical illustrations were generated with BioRender.com. AUC, area under curve; ER, endoplasmic reticulum; MBCD, methyl-beta-cyclodextrin; SRE, sterol regulatory element.

First, we examined if SREBP2 activation by low CO_2_ levels is affected by cellular cholesterol levels. To this end, we exposed cells expressing the SRE reporter to increasing concentrations of MBCD to deplete cholesterol while shifting CO_2_ levels from 5% to 1% (Fig 4A). In line with the above, both sterol depletion under 5% CO_2_ as well as exposure to 1% CO_2_ levels, increased the bioluminescence of the SRE reporter (Fig 4B). Up to 5 mM MBCD, we observed an additive effect in response to 1% CO_2_. Whereas in the presence of higher levels of MBCD, namely, 7 mM, low CO_2_ levels elicited a very minor effect on the activation of the SRE reporter (Fig 4B). The diminished effect of low CO_2_ upon elevated levels of MBCD and likely highly depleted cholesterol levels, raised the possibility that low CO_2_ levels activate SREBP2 in a cholesterol-dependent manner.

To directly examine whether low CO_2_ affected cellular cholesterol levels, we first quantified total cholesterol levels in cells exposed to 5% or 1% CO_2_ for 4 h or upon MBCD treatment. As expected in MBCD treated cells, we observed a marked reduction in total cholesterol levels. By contrast, low or high CO_2_ levels did not show any significant effect on total cellular cholesterol (Figs 4C and S7A, respectively).

SREBP2 is specifically activated in response to changes in ER sterol levels [30]. Furthermore, changes in ER cholesterol levels are sufficient to activate SREBP2 even if total cholesterol levels are unaltered [31]. This prompted us to examine whether CO_2_ alterations specifically affect ER cholesterol levels. Hence, we repeated the above-described experiment, but this time ER membranes were isolated through differential centrifugation, with subsequent sucrose gradient and OptiPrep separation, as previously described [30]. ER membrane free cholesterol and cholesterol ester content were quantified (Fig 4D and 4E). MBCD treatment significantly reduced ER cholesterol (Fig 4D), consistent with previous reports [30,31]. Remarkably, although low CO_2_ levels did not affect total cellular cholesterol, we observed a substantial decrease in ER free cholesterol levels (Figs 4D and S7D). No significant effect on cholesterol esters content was detected (Figs 4E and S7D).

Cholesterol or 25-hydroxylcholesterol supplementation elevates the ER cholesterol pool that acts through SCAP-Insig binding to anchor SREBP2 in the ER and inhibit its activation [32]. To examine the effect of ER cholesterol pools, cells were exposed to low CO_2_ in presence of cholesterol or 25-hydroxycholesterol for 4 h and the expression of cholesterogenic genes was analyzed. The transcriptional response of cholesterogenic genes to low CO_2_ was abolished in presence of cholesterol or hydroxycholesterol, which further supports involvement of cholesterol levels and most likely ER cholesterol on SREBP2 activation under low CO_2_ (Fig 4F).

In summary, our analyses suggest that low CO_2_ specifically alters ER cholesterol, and this effect likely triggers the subsequent processing and activation of SREBP2.

## Discussion

Alterations in CO_2_ levels (hypocapnia or hypercapnia) have been increasingly linked to various pathologies [6,33–35], yet the molecular mechanisms that are implicated in the response to changes in CO_2_ remain elusive.

In the present study, we show that a decrease in CO_2_ levels activate a distinct gene expression program that differs from mere pH changes (e.g., NaOH treatment). These findings support the presence of a specific mechanism that respond to changes in CO_2_ levels. Furthermore, we show that SREBP2 participates in CO_2_ signaling to regulate the expression of its target genes, primarily genes of cholesterol biosynthesis. Of note, CO_2_ is in equilibrium with HCO_3_^-^, hence, we cannot conclude whether the observed cellular response to altered CO_2_ is due to molecular CO_2_ or to changes in bicarbonate levels. Dissecting the effect of CO_2_ per se from associated change in bicarbonates is expected to be challenging in view of their rapid equilibrium in physiological systems. This issue can be potentially addressed by using out-of-equilibrium CO_2_/HCO_3_^-^ solutions [36]. In addition, manipulation of CO_2_ levels in vivo in animal models are extremely challenging due to various homeostatic mechanisms that rapidly act to maintain the equilibrium between CO_2_, bicarbonate, and pH levels.

Interestingly, in pancreatic cancer cells SREBP2 induces the expression of cholesterogenic genes in response to extracellular acidic condition [37]. Consistently, our results show that alkaline conditions per se (i.e., NaOH treatment), unlike exposure to low carbon dioxide levels, elicit a minor effect on the expression of SREBP2 target genes in non-cancerous cells. Hence, it appears that SREBP2 can be activated in response to various stimuli, namely low carbon dioxide levels and acidic conditions. Since low carbon dioxide levels are associated with alkaline and not acidic conditions, it further supports our conclusion that low CO_2_ levels activate SREBP2 through distinct mechanism that is not necessarily pH-related. It is noteworthy that different cell types might respond differently to pH or CO_2_ levels. Previous reports showed that carbon dioxide regulates different signaling pathways such as NFκB, Wnt, and TGFβ signaling, as well as circadian rhythms in different cell types [10,38–40]. We also show that SREBP2 is activated in response to low CO_2_ levels in fibroblast, hepatocytes, and muscles but not in adipocytes. Hence, it is conceivable that the response to CO_2_ is conducted through myriad of signaling pathways, some of which are cell-type specific. Our gene expression analysis identified HIF-1α and YAP as potential candidates that participate in the response to CO_2_. Indeed, hypoxia regulates cholesterol metabolism through HIF-1α [41], yet the involvement of HIF-1α in conjunction with SREBP2 in response to CO_2_ was hitherto never tested. Likewise, recent evidence points towards functional interaction between YAP and SREBP in regulation of lipid metabolism [42,43]; however, its relevance to CO_2_ remains unknown.

Importantly, a role of CO_2_ in the control of cholesterol homeostasis has not been previously reported. Interestingly, cellular cholesterol has been shown in vitro to regulate CO_2_ permeability in different cell types [44,45]. In these studies, each cell type exhibited different CO_2_ permeability rate depending on its cholesterol content [45]. This raises the intriguing possibility of a mechanism whereby changes in CO_2_ levels regulate cholesterol biosynthesis through SREBP2 to regulate cell membrane cholesterol content and control CO_2_ permeability in response to environmental changes.

Although, CO_2_ is generated as a byproduct of cellular enzymatic reactions, CO_2_ is also consumed as a carbon source in the conversion of acetyl-CoA to malonyl-CoA. Acetyl-CoA serves as a key precursor for both fatty acid and cholesterol biosynthesis pathways that are major lipid building blocks for cell membranes. SREBPs control the flux of acetyl-CoA into fatty acid and mevalonate synthetic pathways [46]. Reduction of extracellular CO_2_ might limit the abundance of intracellular CO_2_ and would shift the flux of acetyl-CoA towards cholesterol biosynthesis. This shift in substrate supply may serve to support the increased expression of cholesterogenic enzymes by SREBP2.

At the molecular level, our findings suggest that under physiological growth conditions (i.e., 5% CO_2_), SREBP2 is retained in the ER membrane. Low CO_2_ reduces ER cholesterol levels and triggers SREBP2 translocation from ER to Golgi, where SREBP2 is cleaved. The cleaved, transcriptionally active form of SREBP2 (N-SREBP2) enters the nucleus and activates transcription of cholesterol biosynthetic enzymes through SRE region on the gene promoter (Fig 4G). It remains unclear how CO_2_ modulates the ER lipids composition, and future studies are expected to address the underlying molecular mechanisms.

In summary, we propose that SREBP2 participates in cellular CO_2_ signaling and that SREBP2 regulation of cholesterol levels can be modulated by changes in CO_2_ levels.

## Methods

### Ethics statement

All animal experiments and procedures were conducted in conformity with and approval of the Weizmann Institute Animal Care and Use Committee (IACUC) guidelines, working within the anti-cruelty law (experiments on animals) of 1994 as stated by the Ministry of Health of the Israeli Parliament. Experiments were done in accordance with these specific applications: 05730621–1 and 01700223–2.

### Cell culture

NIH3T3, Hepa1c1 cells were routinely cultured in media A (DMEM with high glucose (01-052-1A, Biological Industries) supplemented with 10% FBS, 100 units/ml penicillin, 100 mg/ml streptomycin, 44 mM NaHCO_3_) at 37°C in a humidified incubator with 5% CO_2_. Mouse tail tip fibroblasts (TTFs) were routinely cultured as previously described [47] with media A containing 20% FBS. Mouse primary muscles were isolated and cultured as previously described [48] with BioAmf2 (Biological Industries Cat # 01-194-1A) and were differentiated in DMEM: F12 (Sigma D6421) supplemented with 2% Horse Serum (04-004-1A, Biological Industries). Fully differentiated fibers were used for the experiment. Mouse white and brown adipocytes were isolated, cultured, and differentiated as previously described [49]. All the experiments were performed in media B (Bicarbonate free DMEM (5×, 01-055-4A Biological Industries) diluted to 1× with deionized water and supplemented with 10% FBS, 4 mM L-Glutamine, 100 units/ml penicillin, and 100 mg/ml streptomycin with 18 mM sodium bicarbonate).

### Reagents and chemicals

Reagents and drugs used are listed in S6 Table. Information regarding dosage and solvents are detailed in the relevant figure legends.

### Plasmids and siRNA transfections

The plasmids pcDNA3.1-2xFLAG-SREBP-2 (#26807), pSynSRE-T-Luc (#60444), pSynSRE-Mut-T-Luc (#60490), pcDNA3-Luciferase (#18964) were purchased from Addgene. The siRNA against mouse SREBP2 (L-050073-01-0010), SCAP (L-040322-01-0010), and control (D-001810-10-50) were purchased from Dharmacon. Briefly, 2.5 × 10^5^ cells were seeded in 3-cm culture dish in media B. For plasmid transfection, in the next day cells were transiently transfected with the 750 ng of the indicated plasmids using jetPRIME (Polyplus) as per the supplier protocol. Alternatively, for siRNA treatment, cells were transfected with 25 nM siRNA using Lipofectamine RNAiMAX (Thermo Fisher Scientific) transfection reagent with 1:3 siRNA/reagent ratio. Cells were analyzed 48 h after transfection by either immunoblot, qPCR, or bioluminescence assays unless indicated otherwise.

### RNA extraction and qPCR analysis

RNA extraction from the cells were performed by TRI-reagent (Sigma) with manufacturer standard protocol. RNA concentration was determined using NanoDrop2000 Spectrophotometer (Thermo Fisher Scientific, USA). RNA integrity was validated using 2200 TapeStation (Agilent, USA). Synthesis of cDNA was performed using qScript cDNA SuperMix (Quanta Biosciences). Real-time PCR measurements were performed using SYBR green primers with LightCycler II machine (Roche) and normalized to the geometrical mean of 3 housekeeping genes (*Rplp0*, *Tbp*, and *Hprt*). Primer sequences are listed in S7 Table.

### RNA sequencing

Bulk MARS-seq libraries [50] were prepared from the mRNA extracted from NIH3T3 cells untreated or exposed to either 1% CO_2_ or 18 mM NaOH under 5% CO_2_ for 0, 2, and 4 h, and subsequently sequenced with high-output 75-base-pair kits (catalogue no. FC-404-2005; Illumina, USA) on a NextSeq 550 Illumina sequencer.

### RNA-sequencing data analysis

Processing of raw sequencing data into read counts was performed via Transcriptome Analysis Pipeline (v.1.10) [51]. In short, reads were trimmed using cutadapt (v.1.15) [52] and mapped to the genome (/shareDB/iGenomes/Mus_musculus/UCSC/mm10/Sequence/STAR_index) using STAR (v.2.5.2b) (default parameters) [53]. The pipeline quantifies the genes annotated in RefSeq that have extended with 1,000 bases towards the 5′ edge and 100 bases towards the 3′ bases. Unique molecular identifier (UMI) counts were done using HTSeq-count (v.0.9.1) in union mode [52]. Normalization of the counts was performed using DESeq2 (v.1.16.1) with the betaPrior = True, cooksCutoff = FALSE, independentFiltering = FALSE parameters [54].

RNA-seq data are available from the GEO database (accession number GSE196294). All other data that support the findings of this study are available upon request.

### Pathway enrichment and upstream transcription factor analysis

Enrichment analysis and upstream transcription factor analysis was performed with Ingenuity Pathway Analysis software QIAGEN IPA (QIAGEN) with default setting.

### Sterol depletion

Sterol depletion from the cells was performed by MBCD (Sigma) treatment. Cells were seeded in media B at density 300,000 cells/3 cm dish. On day 4, cells were washed with PBS twice to remove residual serum and cells were incubated in DMEM (Gibco, 21063–029) without serum, supplemented with either 2 mM MBCD (100 mM stock in water) or vehicle control for 2 h at 37°C and 5% CO_2_. Next, cells were washed twice with PBS and cultured in media B without serum.

### Bioluminescence recordings

Unless indicated otherwise, for bioluminescence recordings cells were seeded in media B at density of 300,000 cells per 3-cm culture dish. Next day, cells were transiently transfected with one of the plasmids containing pSynSRE-T-Luc (750 ng), pSynSRE-Mut-T-Luc (750 ng), or pcDNA3-Luc (500 ng) as detailed above. After 48 h from transfection, the culture medium was replaced with media B supplemented with 100 nM D-Luciferin (Promega, USA) and bioluminescence was recorded continuously with LumiCycle32 recorder (Actimetrics, USA) in a 37°C, 5% CO_2_ incubator. After 24 h (once the luciferase signal reached a stationary phase), CO_2_ levels were shifted to 1%, 10%, or kept at 5% CO_2_ as control. Bioluminescence data were extracted using the LumiCycle Analysis software (Actimetrics, USA). The relative bioluminescence was calculated by normalizing the raw counts to the 10 h pretreatment average value.

### Protein extraction, gel electrophoresis, and immunoblotting

Whole cell lysate was prepared as previously described [9]. For nuclear and cytoplasmic fraction, cell pellets were resuspended in lysis buffer (HEPES 10 mM (pH 7.5), 10 mM KCl, 0.1 mM EDTA, 0.5% Noniodate 40, 1 mM DTT, PMSF 0.5 mM) supplemented with protease inhibitor cocktail (Sigma) and allowed to swell on ice for 15 to 20 min with intermittent mixing. Tubes were vortexed (10 s) to disrupt the cell membrane and then centrifuged at 12,000 g at 4°C for 30 s. The supernatant was stored at −80°C till further use as cytoplasmic extract. The pelleted nuclei were washed twice with 1 ml lysis buffer and was resuspended in nuclear extraction buffer (20 mM HEPES (pH 7.5), 400 mM NaCl, 1 mM EDTA, and 1 mM PMSF) with protease inhibitor cocktail and then incubated on ice for 30 min. Nuclear extracts were collected by centrifugation at 12,000 g for 15 min at 4°C. The protein concentration of the cytoplasmic and nuclear extract was quantified using BCA protein assay kit (Thermo Scientific, USA). Finally, samples were heated at 95°C for 5 min in Laemmli sample buffer and analyzed by SDS-PAGE and immunoblot. SREBP2 antibody that was used in our study (Anti-SREBP2, Clone 22D5, MABS1988, Lot # 3272232, Merck) recognizes the N-terminal region of murine SRE-binding protein 2. Details of the antibodies used are listed under S8 Table.

### ER membrane isolation

Cells were seeded in 15-cm culture dish at density of 2 × 10^6^ cells per dish. On day 4, cells were subjected to different treatments as indicated. Next, cells were washed with cold PBS, scrapped in 2 ml PBS, and collected in 15 ml tube. The suspension was centrifuged at 500 g for 10 min to obtain cell pellet, snap frozen in liquid nitrogen, and stored at −80°C until further use. Isolation of ER membranes was performed with minor modification as previously described [30]. Cell pellet were homogenized with glass dounce (15 to 25 rounds) in cold lysis buffer (50 mM Tris-HCl (pH 7.5), 150 mM NaCl, 15% sucrose) containing protease inhibitor cocktails. A small aliquot of homogenate was stored as whole cell lysate (fraction A) (S7B Fig). The lysates were centrifuged at 3,000 g for 10 min to yield nuclear pellet and supernatant (fraction B). Nuclear pellets were lysed with nuclear extraction buffer (20 mM HEPES (pH 7.5), 400 mM NaCl, 1 mM EDTA, and 1 mM DTT) and stored as fraction C. Further, the supernatant was diluted to 3 ml with lysis buffer and loaded on discontinuous sucrose gradient which was set in SW41 tube (Beckman) by overlaying the following sucrose solutions all in the above lysis buffer: 2 ml 45%, 4 ml 30%, 3 ml of the diluted supernatant in 15% sucrose, and 1 ml 7.5%. The tubes were ultra-centrifuged in SW41Ti rotor (Beckman) at 100,000 g for 60 min and allowed to slow down without application of a break. The 2 bands of membranes were clearly visible, upper light membrane fraction (Interphase between 15% and 7.5%) were collected and marked as fraction D and heavy membrane fraction (interphase between 45% and 30% sucrose) were collected in another tube as fraction E. The collected fractions at each stage were analyzed by immunoblot with relevant organelle markers as indicated (S7C Fig). Further, purification of heavy membrane fraction (fraction E) was performed with OptiPrep-Density gradient medium (Sigma). Fraction E from the above sucrose gradient was loaded at the bottom of SW41 tube and subsequently, overlaid with dilutions of OptiPrep-Density gradient medium. Discontinuous OptiPrep gradient was generated by underlying in sequence form bottom to top—1 ml fraction E, 2.5 ml each of 25%, 23%, 21%, 19% OptiPrep media diluted in ice cold tris-buffer (50 mM Tris-HCl (pH 7.5), 150 mM NaCl) and equilibrate for 2 h at 4°C. After incubation, tubes were ultra-centrifuged at a speed of 110,000 g for 120 min. After centrifugation, OptiPrep fractions were collected from top to bottom of the tube (approximately 900 μl each fraction) and fractions were run on the SDS-PAGE with marker protein for ER membrane and the fraction showing no organelle contamination (fraction no 5) was used for lipidomic analysis.

### ER-lipidomic analysis

Cholesterol and cholesterol ester were identified and quantified using multi-dimensional mass spectrometry-based shotgun lipidomic analysis [55]. In brief, each 300 μl ER suspension sample was accurately transferred to a disposable glass culture test tube. A pre-mixture of internal standards (IS) was added prior to conducting lipid extraction for quantification of the targeted lipid species based on the protein content of individual ER suspension. Lipid extraction was performed using a modified Bligh and Dyer procedure [56], and each lipid extract was reconstituted in chloroform:methanol (1:1, *v*:*v*) at a volume of 100 μl/300 μl ER suspension samples.

For shotgun lipidomics, lipid extract was further diluted to a final concentration of approximately 500 fmol total lipids per μl. Mass spectrometric analysis was performed on a triple quadrupole mass spectrometer (TSQ Altis, Thermo Fisher Scientific, USA) and a Q Exactive mass spectrometer (Thermo Scientific, USA), both of which were equipped with an automated nanospray device (TriVersa NanoMate, Advion Bioscience Ltd., Ithaca, NY) as described [57]. Identification and quantification of cholesterol and cholesterol ester were performed using an automated software program [58,59]. Data processing (e.g., ion peak selection, baseline correction, data transfer, peak intensity comparison, and quantitation) was performed as described [59]. The results were normalized to volume of ER suspension (pmol/100 μl ER suspension).

### Total cholesterol quantification

Lipid extraction and cholesterol quantification were performed using a Total Cholesterol Assay Kit Fluorometrically (Cell Biolabs, STA-390) according to the manufacturer’s protocol.

### Statistics

All the statistical analyses were performed using Excel, Python, and GraphPad prism (Version 9.1.0.221). Specific information on sample sizes, statistical significance, and variance measures is provided in the relevant figure legends. Significantly expressed genes are defined based on the difference in expression between each of the time points (i.e., T0, T2, T4) per condition, based on the following criteria: P adj. < = 0.05, |log2FC| > = 1, baseMean > = 5. Normalized data of significant genes in each of the conditions was clustered using Python’s scikit-learn KMeans function. PCA analysis was performed using R’s prcomp function (scale = TRUE) of stats package.

## Supporting information

S1 Fig Transcriptional response to low CO_2_ levels compared to NaOH treatment. **(A)** Bar plot representing the number of significant genes (see Methods) that were up- or down-regulated in response to 1% CO_2_ or 18 mM NaOH after 2 or 4 h exposure. **(B)** Venn diagrams representing the number of genes that significantly responded to 1% CO_2_ or 18 mM NaOH after 2 or 4 h and their overlaps. The data underlying the graphs shown in the figure is included in S1 Data.
(TIF)

S2 Fig Low CO_2_ levels induce the expression of cholesterol biosynthesis related genes. **(A)** Quantitative PCR analysis of cholesterogenic gene expression levels from mouse Hepa1c1, primary tail tip fibroblasts (TTF), mouse primary muscles, white adipocytes (WAT), and brown adipocytes (BAT) cultured either at 5% CO_2_ or 1% CO_2_ for 4 h (mean ± SE, *n* = 3 biological replicates for Hepa1c1, *n* = 3 biological replicates per condition for TTF, muscles, WAT and BAT, ***P < 0.001, **P < 0.01, *P < 0.05, nonsignificant (ns), two-way ANOVA with Bonferroni’s multiple comparisons test). **(B)** Quantitative PCR analysis of cholesterogenic gene expression levels from NIH3T3 cultured either at 5% CO_2_ or 10% CO_2_ for 2 or 4 h (mean ± SE, *n* = 3 biological replicates for each time point per condition, ***P < 0.001, **P < 0.01, *P < 0.05, nonsignificant (ns), two-way ANOVA with Bonferroni’s multiple comparisons test). The data underlying the graphs shown in the figure is included in S1 Data.
(TIF)

S3 Fig Comparative analysis of gene expression data from cells exposed to different CO_2_ levels. **(A, B)** Gene expression data (Phelan and colleagues) of THP-1 monocytes exposed to 10% CO_2_ for 4 h was compared with data of NIH3T3 exposed to 1% CO_2_ for 2 h and 4 h. **(A)** A Venn diagram presentation of the overlap in the responsive genes from both datasets. **(B)** A bar graph presentation of the transcriptional response of common genes (28 for 2 h and 47 for 4 h). The data underlying the graphs shown in the figure is included in S1 Data.
(TIF)

S4 Fig SREBP2 is activated in response to low CO_2_ levels but not in response to alkaline conditions. **(A)** Immunoblot of total cell extracts from NIH3T3 cells exposed to either 5% CO_2_ or 18 mM NaOH for 0, 2, or 4 h. p—SREBP2 precursor (approximately 126 kD); c—SREBP2 cleaved form (approximately 68 kD); (pooled sample of *n* = 3 biological replicates). **(B)** Quantitative PCR analysis for the expression levels of CO_2_ responsive genes from control (siNTC) or SREBP2 silenced (siSREBP2) NIH3T3 cells exposed to 5% or 1% CO_2_ for 4 h (mean ± SE, *n* = 3 biological replicates per condition, ***P < 0.001, **P < 0.01, nonsignificant (ns), two-way ANOVA with Bonferroni’s multiple comparisons test). The data underlying the graphs shown in the figure is included in S1 Data.
(TIF)

S5 Fig CO_2_ levels modulate the response of an SRE bioluminescence reporter. **(A)** Bioluminescence recordings from NIH3T3 cells transfected with WT SRE-Luc, or control vector (CMV-Luc), and exposed to either 5%, 1%, or 10% CO_2_, arrow indicates the shift in CO_2_ levels (mean ± SE, *n* = 3 biological replicate per condition, AUC for SRE Luc 5%, 1% CO_2_ 0.77 ± 0.01, 2.6 ± 0.1, *P* < 0.0001 and 5%, 10% CO_2_ 1.04 ± 0.005, 0.65 ± 0.02, *P* < 0.0001; AUC for CMV Luc 5%, 1% CO_2_ 0.2 ± 0.002, 0.19 ± 0.005, ns and 5%, 10% CO_2_ 0.65 ± 0.02, 0.68 ± 0.01, ns, nonsignificant (ns), two-sided Student’s *t* test). **(B)** Bioluminescence recordings from NIH3T3 cells transfected with WT SRE-Luc exposed either to constant 5% or interchangeable 5% to 1% CO_2_ levels, blue mark represents 5%, and red mark indicates 1% CO_2_ levels (mean ± SE, *n* = 3 biological replicate per condition). The data underlying the graphs shown in the figure is included in S1 Data.
(TIF)

S6 Fig Systematic dissection of SREBP2 pathway activation in response to low CO_2_ levels. **(A)** Schematic representation of the SREBP2 pathway, specifying interventions applied at different stages in the following experiments. **(B, C)** Immunoblots of total cell lysates from NIH3T3 cells either non-transfected or transfected with 2X-FLAG tagged N-SREBP2. Cells were either sterol-depleted with methyl-beta-cyclodextrin (MBCD) or CO_2_ treated for 4 h (pooled sample from *n* = 3 biological replicates). **(D)** Immunoblot of total cell lysates from NIH3T3 cells exposed to different CO_2_ levels in presence of DMSO or fatostatin (20 μm) for 4 h (pooled sample from *n* = 3 biological replicates). **(E)** Quantitative PCR analysis of cholestrogenic gene expression levels from cells as in (D), (mean ± SE, *n* = 3 biological replicates for each time point per condition, ***P < 0.001, nonsignificant (ns), two-way ANOVA with Bonferroni’s multiple comparisons test). **(F)** Quantitative PCR analysis of cholestrogenic gene expression levels from NIH3T3 cells silenced for SCAP (siSCAP) or control siRNA (siNTC) upon exposure to either 5% or 1% CO_2_ levels for 4 h (mean ± SE, *n* = 3 biological replicates for each time point per condition, ***P < 0.001, nonsignificant (ns), two-way ANOVA with Bonferroni’s multiple comparisons test). The data underlying the graphs shown in the figure is included in S1 Data.
(TIF)

S7 Fig Exposure to low CO_2_ levels decreases ER cholesterol levels. **(A)** Total cholesterol quantification (with fluorometric assay kit) in NIH3T3 cells exposed to 10% CO_2_ levels for 4 h (mean ± SE, *n* = 3 biological replicates per condition, nonsignificant (ns), two-sided Student’s *t* test). **(B)** Flow chart representing the different steps taken for ER-membrane isolation by sucrose gradient. **(C)** Immunoblot analysis of organelle protein markers in each fraction throughout the isolation process (as detailed in panel B). **(D)** The free cholesterol and cholesterol ester levels in the ER membrane from NIH3T3 cells depleted with sterols for 2 h or exposed to different CO_2_ levels for 4 h were quantified with shotgun lipidomics analysis (see S5 Table) (mean ± SE, *n* = 3 independent experiments, two-sided Student’s *t* test). The data is from Fig 4C and 4D corrected to ER protein amounts. The data underlying the graphs shown in the figure is included in S1 Data.
(TIF)

S1 Table Transcriptional response to low CO_2_ and NaOH (separate file). List of differentially expressed genes from NIH3T3 cells treated with either low CO_2_ or 18 mM NaOH for 2 h and 4 h.
(XLSX)

S2 Table K-Mean cluster analysis (separate file). List of genes that were categorized the clusters for described in Fig 2 for NIH3T3 cells treated with either low CO_2_ or 18 mM NaOH for 2 h and 4 h.
(XLSX)

S3 Table Pathway enrichment analysis (separate file). Full list of processes enriched in different clusters (as in Fig 2A) in NIH3T3 cells exposed to 5%, 1% CO_2_ or treated with 18 mM NaOH for 2 h and 4 h.
(XLSX)

S4 Table Upstream gene expression regulator analysis (separate file). Full list of upstream gene expression regulators in the different clusters (as in Fig 3A and 3B) in NIH3T3 cells exposed to either low CO_2_ or NaOH for 2 h and 4 h.
(XLSX)

S5 Table ER lipidomics analysis (separate file). Measurement of cholesterol and cholesterol ester different classes of ER lipids with shot gun lipidomics in ER fractions isolated from NIH3T3 cells either depleted for sterols for exposed to 5% CO_2_ or 1% CO_2_ for 4 h.
(XLSX)

S6 Table Chemicals and reagents (separate file). List of reagents and chemicals that were used in the study including their manufactures and catalogue numbers.
(XLSX)

S7 Table List of primers used for quantitative real-time PCR (separate file). Primers used for quantitative PCR analysis are listed in the table.
(XLSX)

S8 Table List of antibodies (separate file). Antibodies and the dilutions used for immunoblot analysis are detailed in the table.
(XLSX)

S1 Data Original data for the different graphs (separate file). Each tab includes data for individual panels of main and supplementary figures as referred to in the figure captions.
(XLSX)

S1 Raw Images Original blots (separate file). The file contains the original and unprocessed blots that are presented in main and supplementary figures.
(PPTX)

## Abbreviations

COPD: chronic obstructive pulmonary disease
ER: endoplasmic reticulum
HIF: hypoxia-inducible factor
INSIG: insulin-induced gene
MBCD: methyl-beta-cyclodextrin
OSA: obstructive sleep apnea
PCA: principal component analysis
SCAP: SREBP cleavage-activating protein
SRE: sterol regulatory element
TTF: tail tip fibroblast
UMI: unique molecular identifier
