# Supplementary figures and images for: Carbon dioxide regulates cholesterol levels through SREBP2
Source: PLoS Biol. 2023 Nov 15;21(11):e3002367. doi: 10.1371/journal.pbio.3002367 (PMC10651039; doi:10.1371/journal.pbio.3002367)

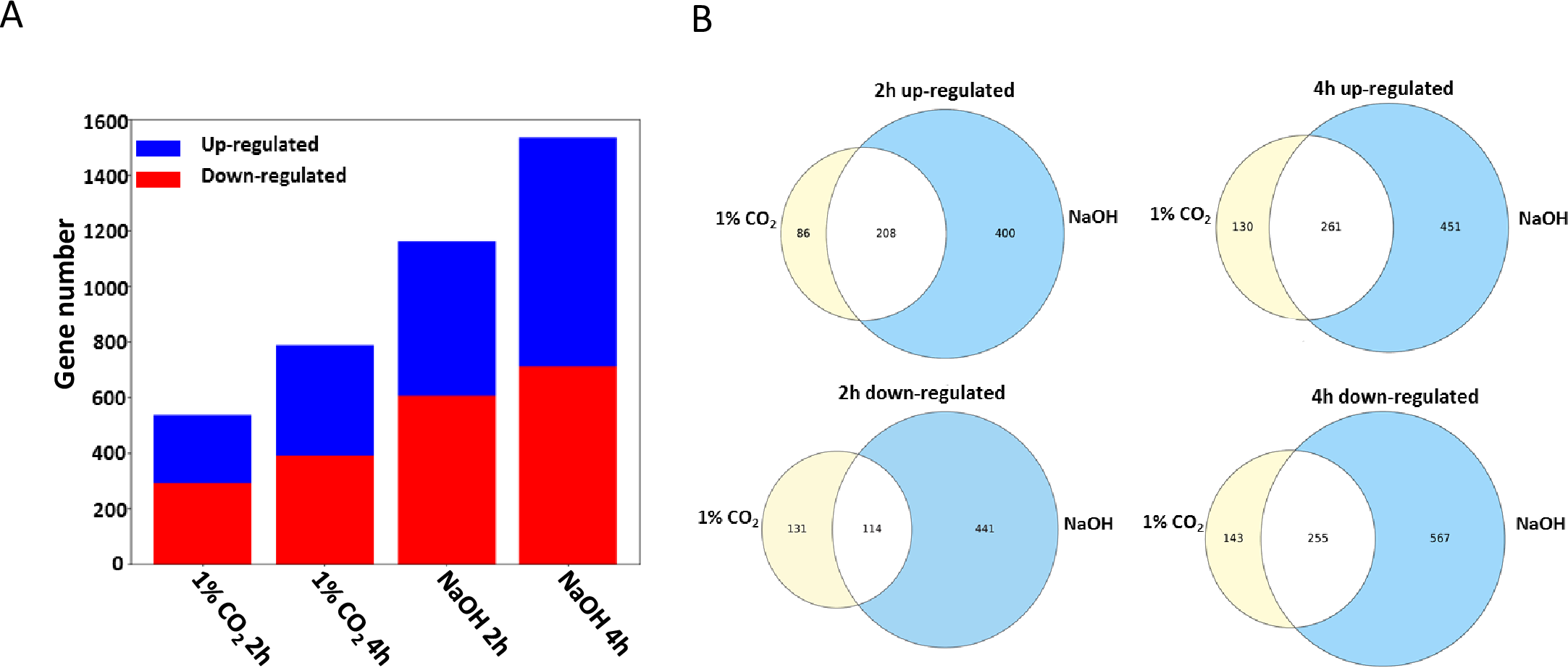

Supplement: S1 Fig — (A) Bar plot representing the number of significant genes (see Methods) that were up- or down-regulated in response to 1% CO2 or 18 mM NaOH after 2 or 4 h exposure. (B) Venn diagrams representing the number of genes that significantly responded to 1% CO2 or 18 mM NaOH after 2 or 4 h and their overlaps. The data underlying the graphs shown in the figure is included in S1 Data. (TIF) [file pbio.3002367.s001.tif]

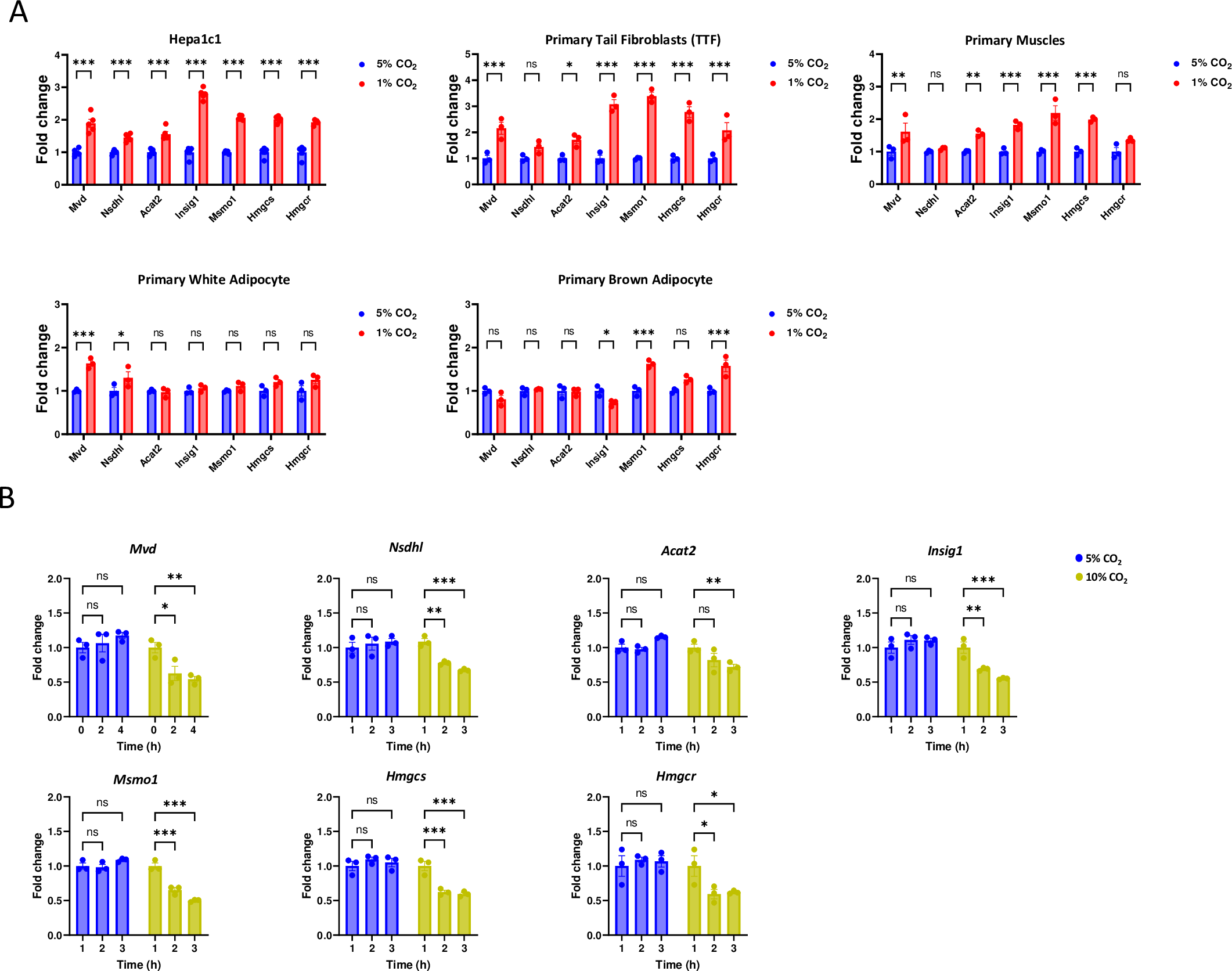

Supplement: S2 Fig — (A) Quantitative PCR analysis of cholesterogenic gene expression levels from mouse Hepa1c1, primary tail tip fibroblasts (TTF), mouse primary muscles, white adipocytes (WAT), and brown adipocytes (BAT) cultured either at 5% CO2 or 1% CO2 for 4 h (mean ± SE, n = 3 biological replicates for Hepa1c1, n = 3 biological replicates per condition for TTF, muscles, WAT and BAT, ***P < 0.001, **P < 0.01, *P < 0.05, nonsignificant (ns), two-way ANOVA with Bonferroni’s multiple comparisons test). (B) Quantitative PCR analysis of cholesterogenic gene expression levels from NIH3T3 cultured either at 5% CO2 or 10% CO2 for 2 or 4 h (mean ± SE, n = 3 biological replicates for each time point per condition, ***P < 0.001, **P < 0.01, *P < 0.05, nonsignificant (ns), two-way ANOVA with Bonferroni’s multiple comparisons test). The data underlying the graphs shown in the figure is included in S1 Data. (TIF) [file pbio.3002367.s002.tif]

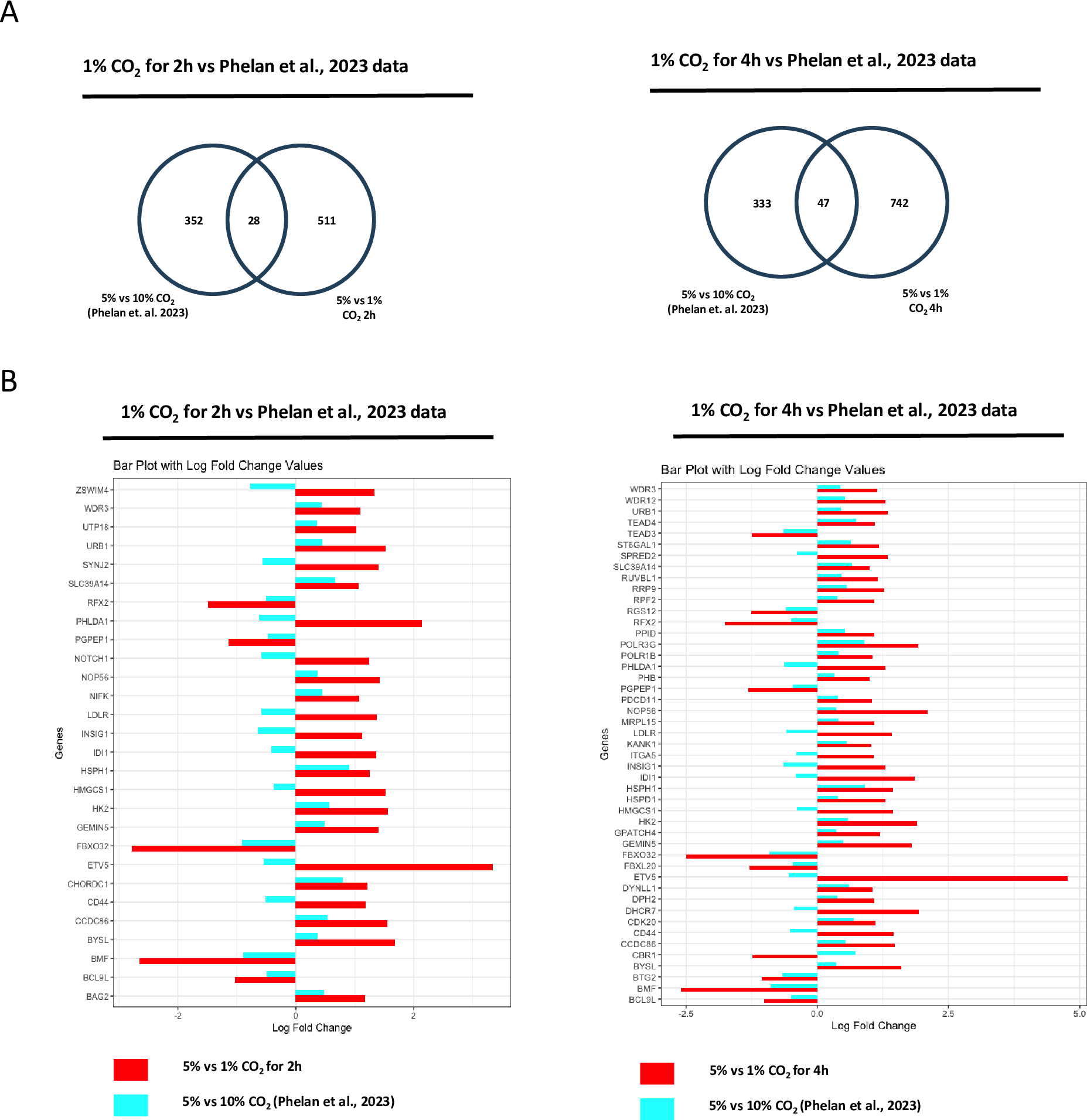

Supplement: S3 Fig — (A, B) Gene expression data (Phelan and colleagues) of THP-1 monocytes exposed to 10% CO2 for 4 h was compared with data of NIH3T3 exposed to 1% CO2 for 2 h and 4 h. (A) A Venn diagram presentation of the overlap in the responsive genes from both datasets. (B) A bar graph presentation of the transcriptional response of common genes (28 for 2 h and 47 for 4 h). The data underlying the graphs shown in the figure is included in S1 Data. (TIF) [file pbio.3002367.s003.tif]

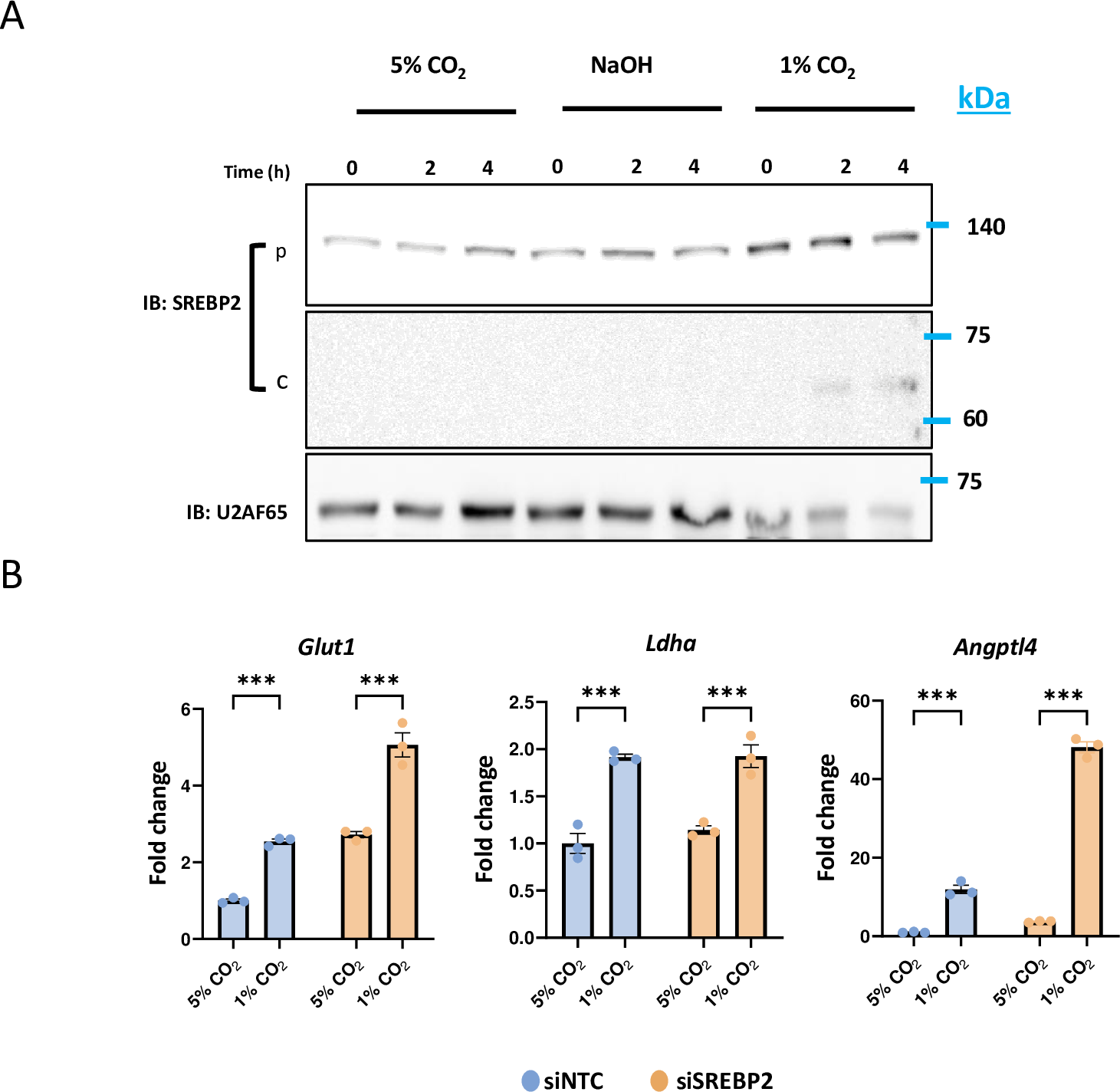

Supplement: S4 Fig — (A) Immunoblot of total cell extracts from NIH3T3 cells exposed to either 5% CO2 or 18 mM NaOH for 0, 2, or 4 h. p—SREBP2 precursor (approximately 126 kD); c—SREBP2 cleaved form (approximately 68 kD); (pooled sample of n = 3 biological replicates). (B) Quantitative PCR analysis for the expression levels of CO2 responsive genes from control (siNTC) or SREBP2 silenced (siSREBP2) NIH3T3 cells exposed to 5% or 1% CO2 for 4 h (mean ± SE, n = 3 biological replicates per condition, ***P < 0.001, **P < 0.01, nonsignificant (ns), two-way ANOVA with Bonferroni’s multiple comparisons test). The data underlying the graphs shown in the figure is included in S1 Data. (TIF) [file pbio.3002367.s004.tif]

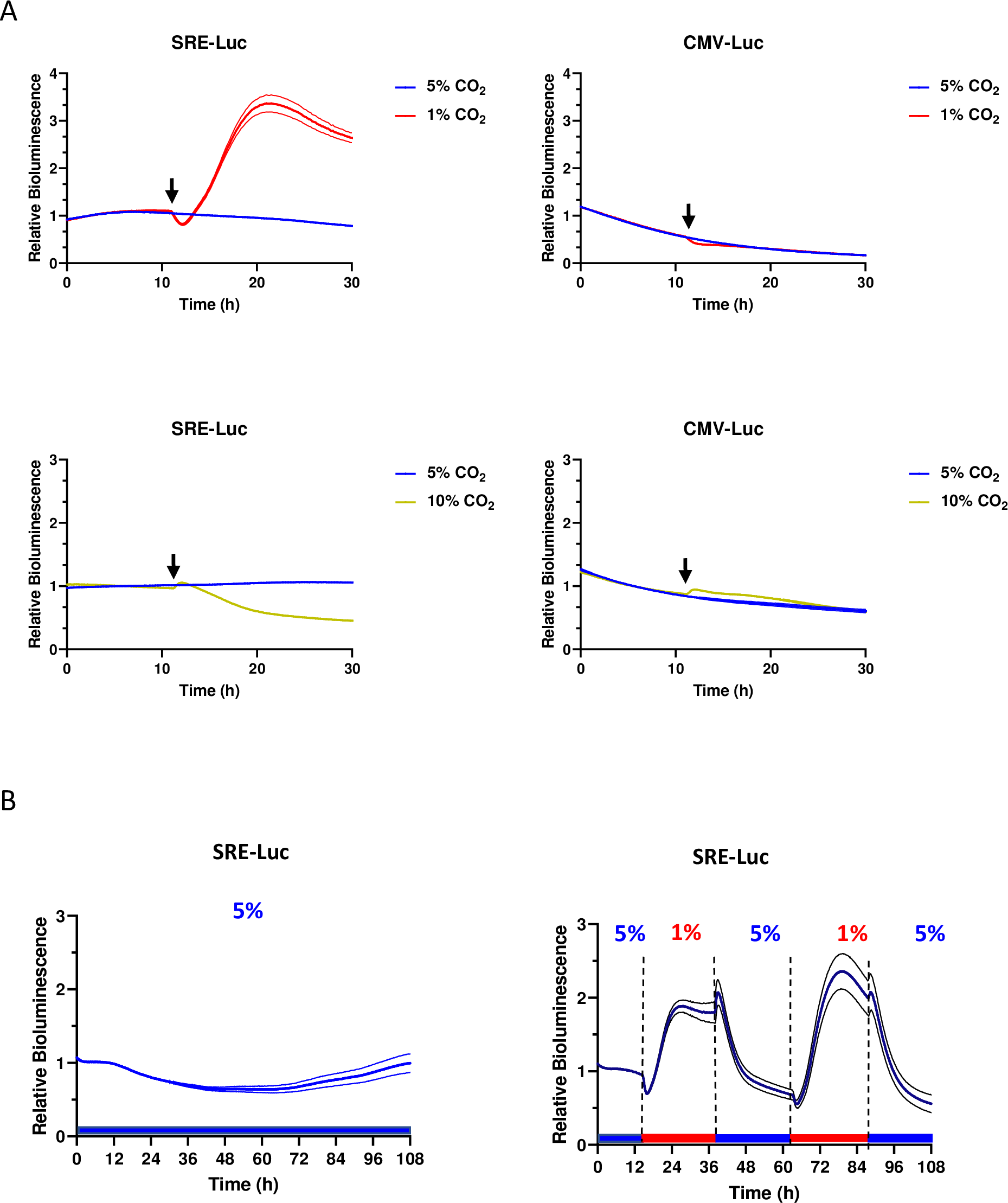

Supplement: S5 Fig — (A) Bioluminescence recordings from NIH3T3 cells transfected with WT SRE-Luc, or control vector (CMV-Luc), and exposed to either 5%, 1%, or 10% CO2, arrow indicates the shift in CO2 levels (mean ± SE, n = 3 biological replicate per condition, AUC for SRE Luc 5%, 1% CO2 0.77 ± 0.01, 2.6 ± 0.1, P < 0.0001 and 5%, 10% CO2 1.04 ± 0.005, 0.65 ± 0.02, P < 0.0001; AUC for CMV Luc 5%, 1% CO2 0.2 ± 0.002, 0.19 ± 0.005, ns and 5%, 10% CO2 0.65 ± 0.02, 0.68 ± 0.01, ns, nonsignificant (ns), two-sided Student’s t test). (B) Bioluminescence recordings from NIH3T3 cells transfected with WT SRE-Luc exposed either to constant 5% or interchangeable 5% to 1% CO2 levels, blue mark represents 5%, and red mark indicates 1% CO2 levels (mean ± SE, n = 3 biological replicate per condition). The data underlying the graphs shown in the figure is included in S1 Data. (TIF) [file pbio.3002367.s005.tif]

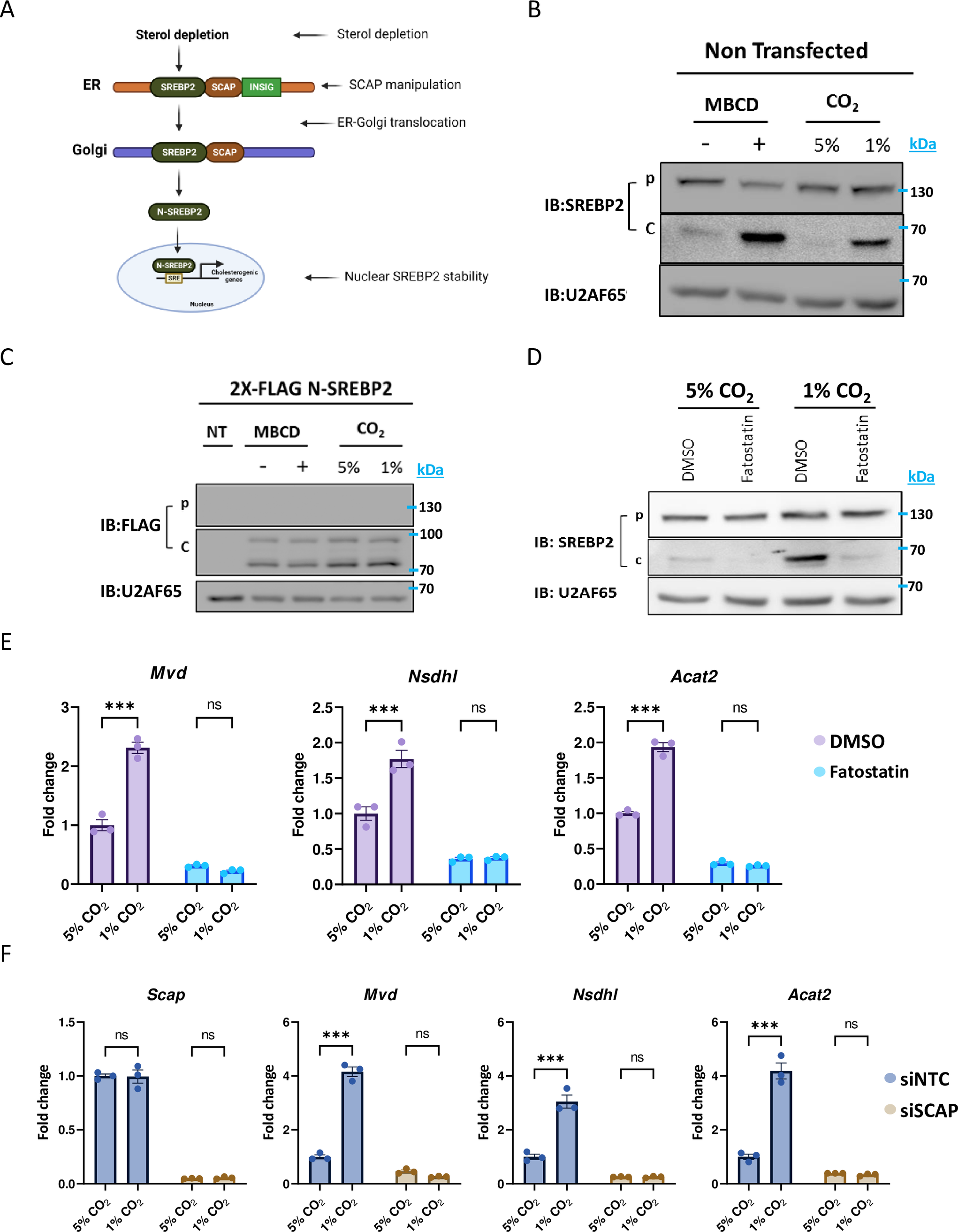

Supplement: S6 Fig — (A) Schematic representation of the SREBP2 pathway, specifying interventions applied at different stages in the following experiments. (B, C) Immunoblots of total cell lysates from NIH3T3 cells either non-transfected or transfected with 2X-FLAG tagged N-SREBP2. Cells were either sterol-depleted with methyl-beta-cyclodextrin (MBCD) or CO2 treated for 4 h (pooled sample from n = 3 biological replicates). (D) Immunoblot of total cell lysates from NIH3T3 cells exposed to different CO2 levels in presence of DMSO or fatostatin (20 μm) for 4 h (pooled sample from n = 3 biological replicates). (E) Quantitative PCR analysis of cholestrogenic gene expression levels from cells as in (D), (mean ± SE, n = 3 biological replicates for each time point per condition, ***P < 0.001, nonsignificant (ns), two-way ANOVA with Bonferroni’s multiple comparisons test). (F) Quantitative PCR analysis of cholestrogenic gene expression levels from NIH3T3 cells silenced for SCAP (siSCAP) or control siRNA (siNTC) upon exposure to either 5% or 1% CO2 levels for 4 h (mean ± SE, n = 3 biological replicates for each time point per condition, ***P < 0.001, nonsignificant (ns), two-way ANOVA with Bonferroni’s multiple comparisons test). The data underlying the graphs shown in the figure is included in S1 Data. (TIF) [file pbio.3002367.s006.tif]

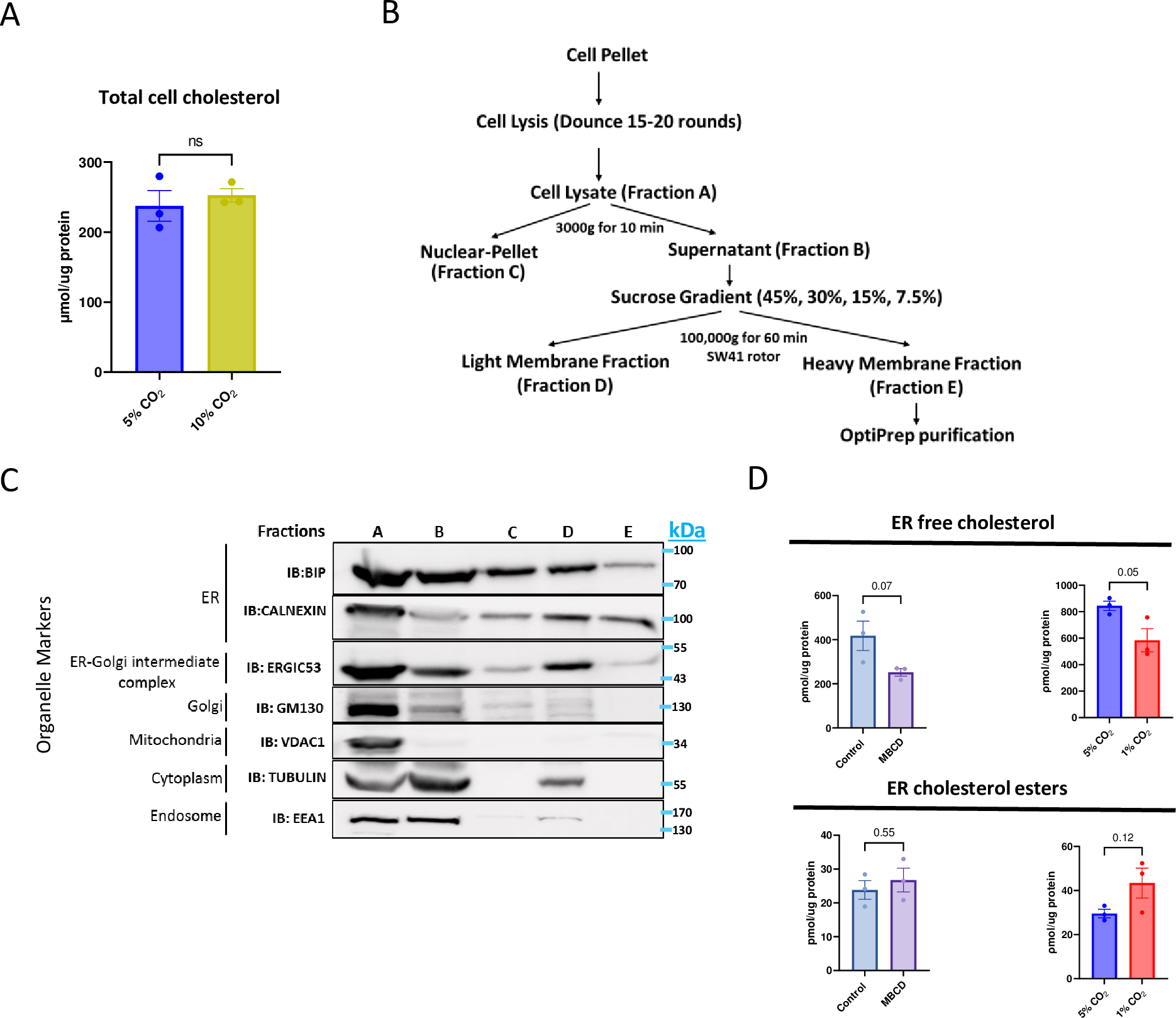

Supplement: S7 Fig — (A) Total cholesterol quantification (with fluorometric assay kit) in NIH3T3 cells exposed to 10% CO2 levels for 4 h (mean ± SE, n = 3 biological replicates per condition, nonsignificant (ns), two-sided Student’s t test). (B) Flow chart representing the different steps taken for ER-membrane isolation by sucrose gradient. (C) Immunoblot analysis of organelle protein markers in each fraction throughout the isolation process (as detailed in panel B). (D) The free cholesterol and cholesterol ester levels in the ER membrane from NIH3T3 cells depleted with sterols for 2 h or exposed to different CO2 levels for 4 h were quantified with shotgun lipidomics analysis (see S5 Table) (mean ± SE, n = 3 independent experiments, two-sided Student’s t test). The data is from Fig 4C and 4D corrected to ER protein amounts. The data underlying the graphs shown in the figure is included in S1 Data. (TIF) [file pbio.3002367.s007.tif]
