# Supplementary material for: Carbon dioxide regulates cholesterol levels through SREBP2
Source: PLoS Biol. 2023 Nov 15;21(11):e3002367. doi: 10.1371/journal.pbio.3002367 (PMC10651039; doi:10.1371/journal.pbio.3002367)
Supplement: S1 Raw Images — The file contains the original and unprocessed blots that are presented in main and supplementary figures. (PPTX) [file pbio.3002367.s017.pptx]

## Slide 1
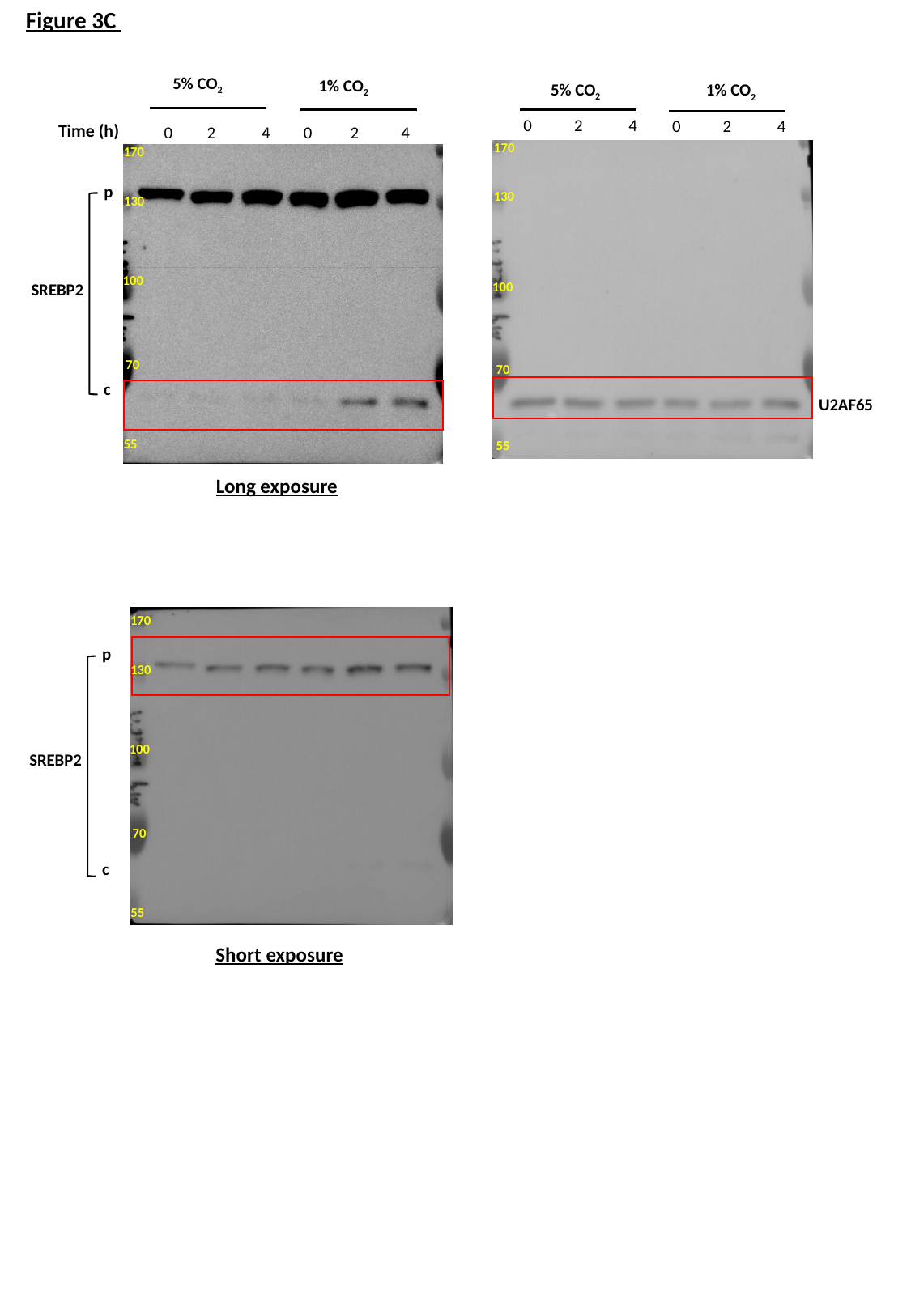

Figure 3C
5% CO2
1% CO2
5% CO2
1% CO2
 0 2 4
0 2 4
Time (h)
0 2 4
0 2 4
170
130
100
70
55
170
130
100
70
55
p
SREBP2
c
U2AF65
Long exposure
170
130
100
70
55
p
SREBP2
c
Short exposure

## Slide 2
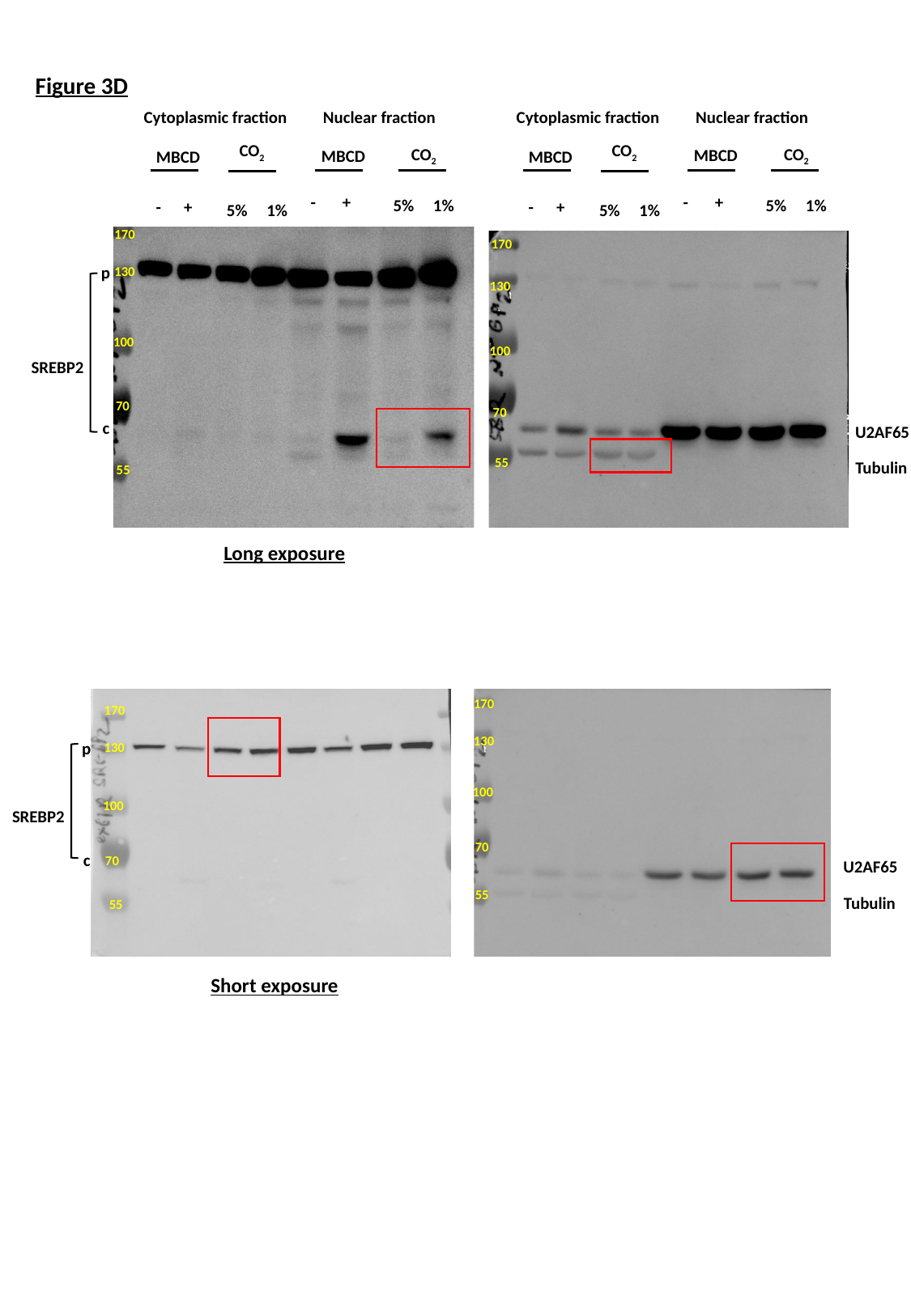

Figure 3D
Cytoplasmic fraction
Nuclear fraction
CO2
CO2
MBCD
MBCD
- +
5% 1%
- +
5% 1%
Cytoplasmic fraction
Nuclear fraction
CO2
CO2
MBCD
MBCD
- +
5% 1%
- +
5% 1%
170
130
100
70
55
170
130
100
70
55
p
SREBP2
c
U2AF65
Tubulin
Long exposure
170
130
100
70
55
170
130
100
70
55
p
SREBP2
c
U2AF65
Tubulin
Short exposure

## Slide 3
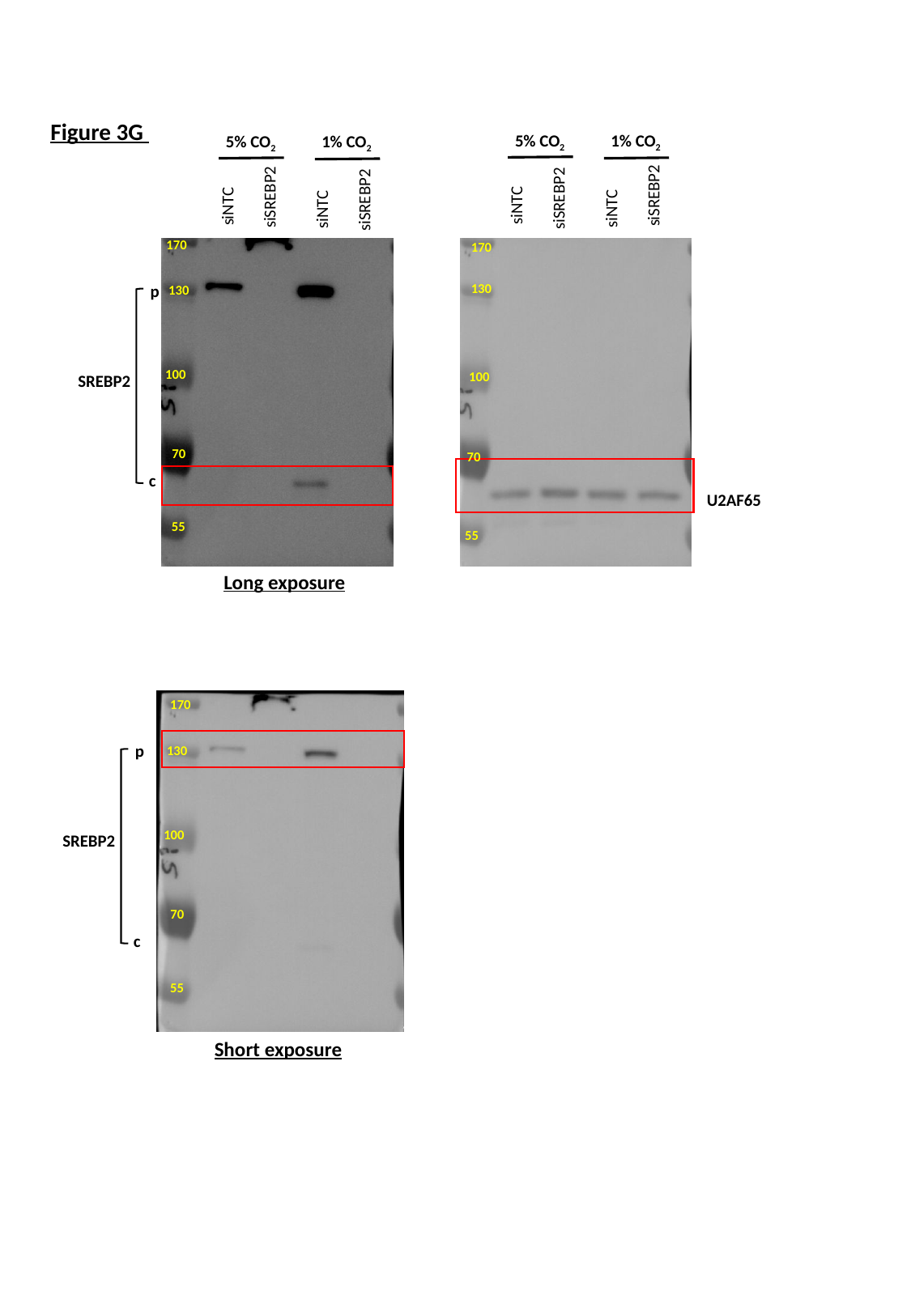

Figure 3G
5% CO2
1% CO2
5% CO2
1% CO2
siSREBP2
siSREBP2
siSREBP2
siSREBP2
siNTC
siNTC
siNTC
siNTC
170
130
100
70
55
170
130
100
70
55
p
SREBP2
c
U2AF65
Long exposure
170
130
100
70
55
p
SREBP2
c
Short exposure

## Slide 4
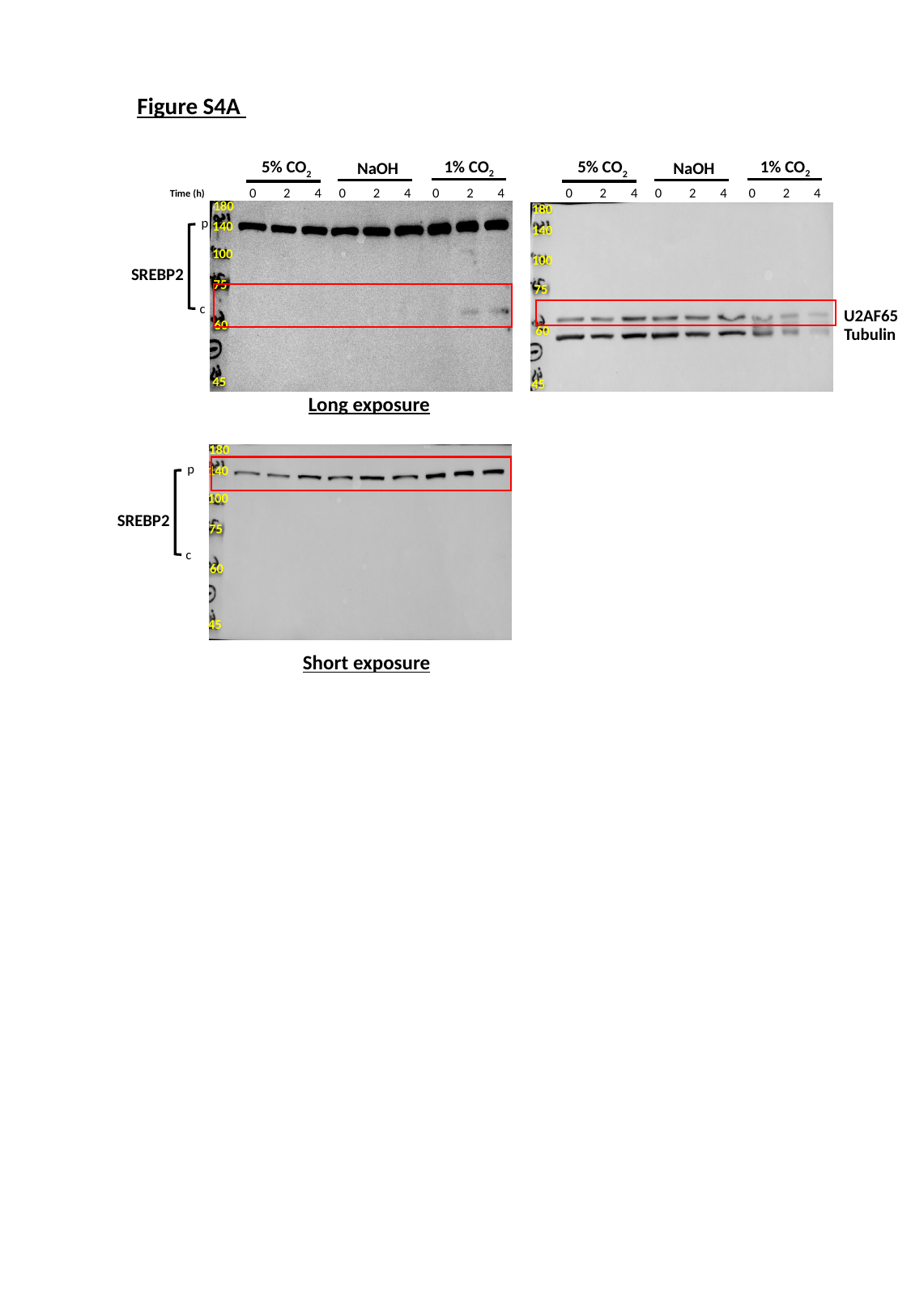

Figure S4A
1% CO2
5% CO2
NaOH
0 2 4
0 2 4
0 2 4
Time (h)
1% CO2
5% CO2
NaOH
0 2 4
0 2 4
0 2 4
180
180
p
SREBP2
c
140
140
100
100
75
75
U2AF65
.
60
60
Tubulin
45
45
Long exposure
180
p
SREBP2
c
140
100
75
60
45
Short exposure

## Slide 5
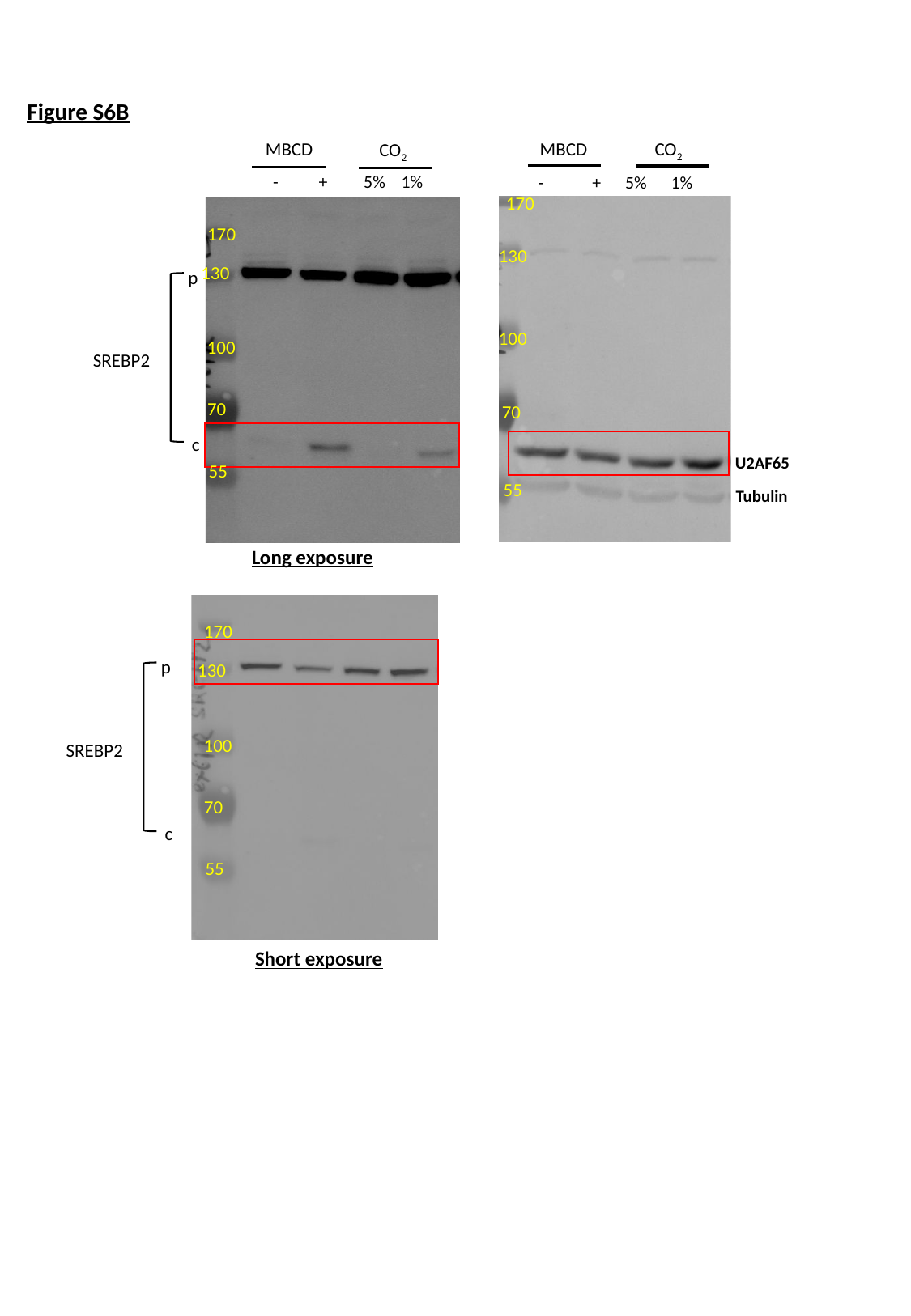

Figure S6B
CO2
MBCD
MBCD
CO2
- + 5% 1%
- + 5% 1%
170
130
100
70
55
170
130
100
70
55
p
SREBP2
c
U2AF65
Tubulin
Long exposure
170
130
100
70
55
p
SREBP2
c
Short exposure

## Slide 6
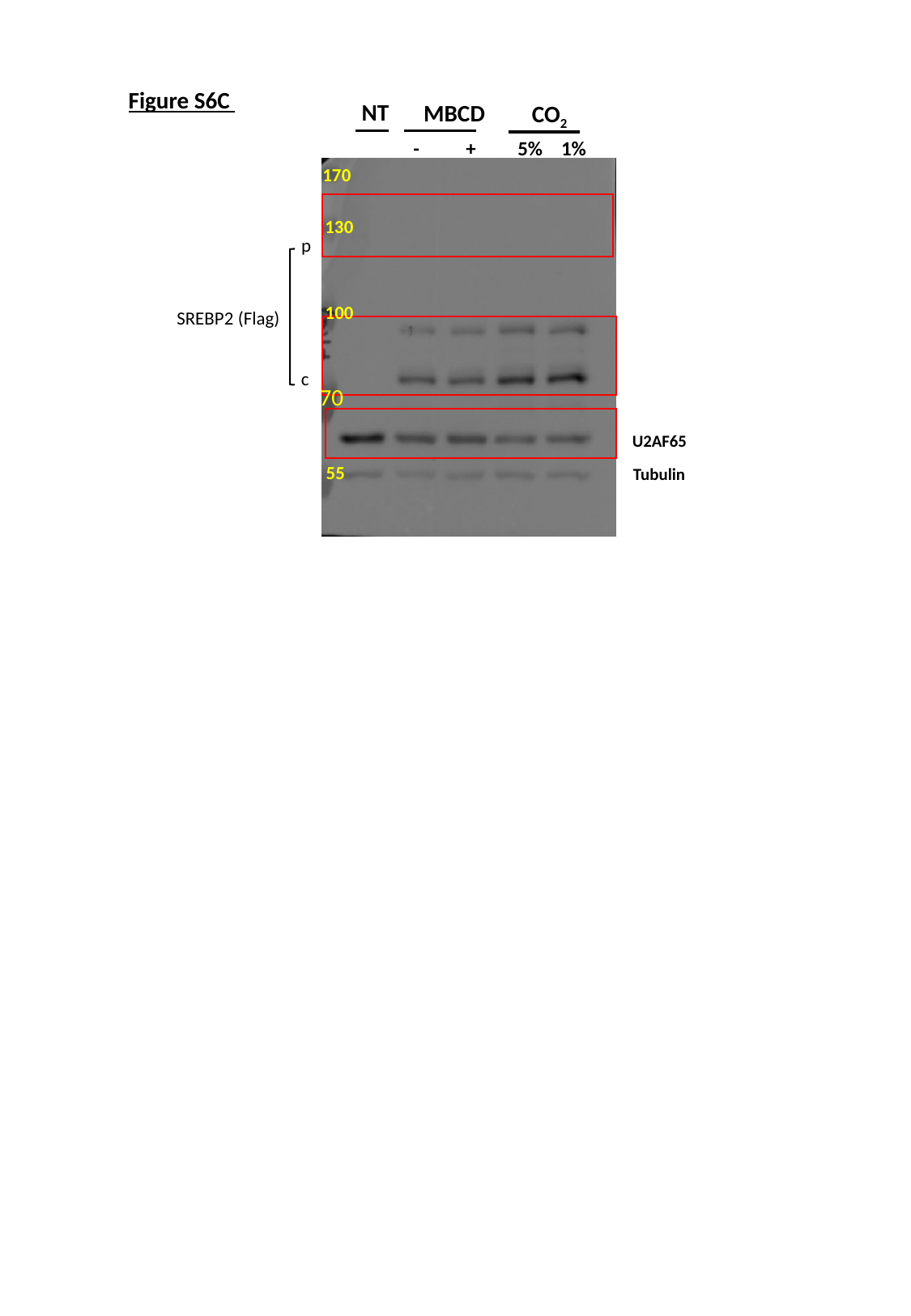

Figure S6C
NT
MBCD
CO2
- + 5% 1%
170
130
100
70
55
p
SREBP2 (Flag)
c
U2AF65
Tubulin

## Slide 7
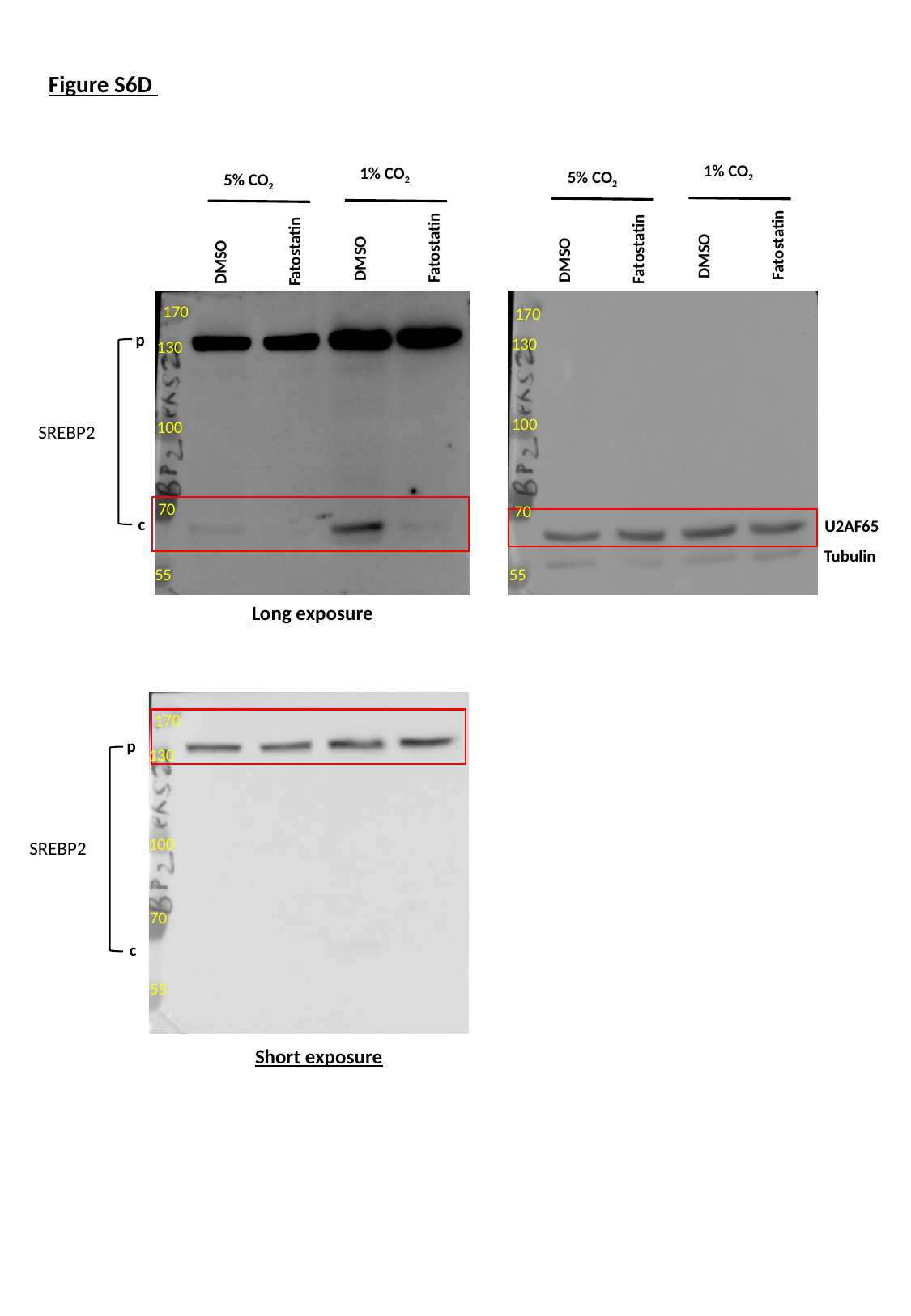

Figure S6D
1% CO2
1% CO2
5% CO2
5% CO2
Fatostatin
Fatostatin
DMSO
DMSO
Fatostatin
Fatostatin
DMSO
DMSO
170
170
p
SREBP2
c
130
130
100
100
70
70
U2AF65
Tubulin
55
55
Long exposure
170
p
SREBP2
c
130
100
70
55
Short exposure

## Slide 8
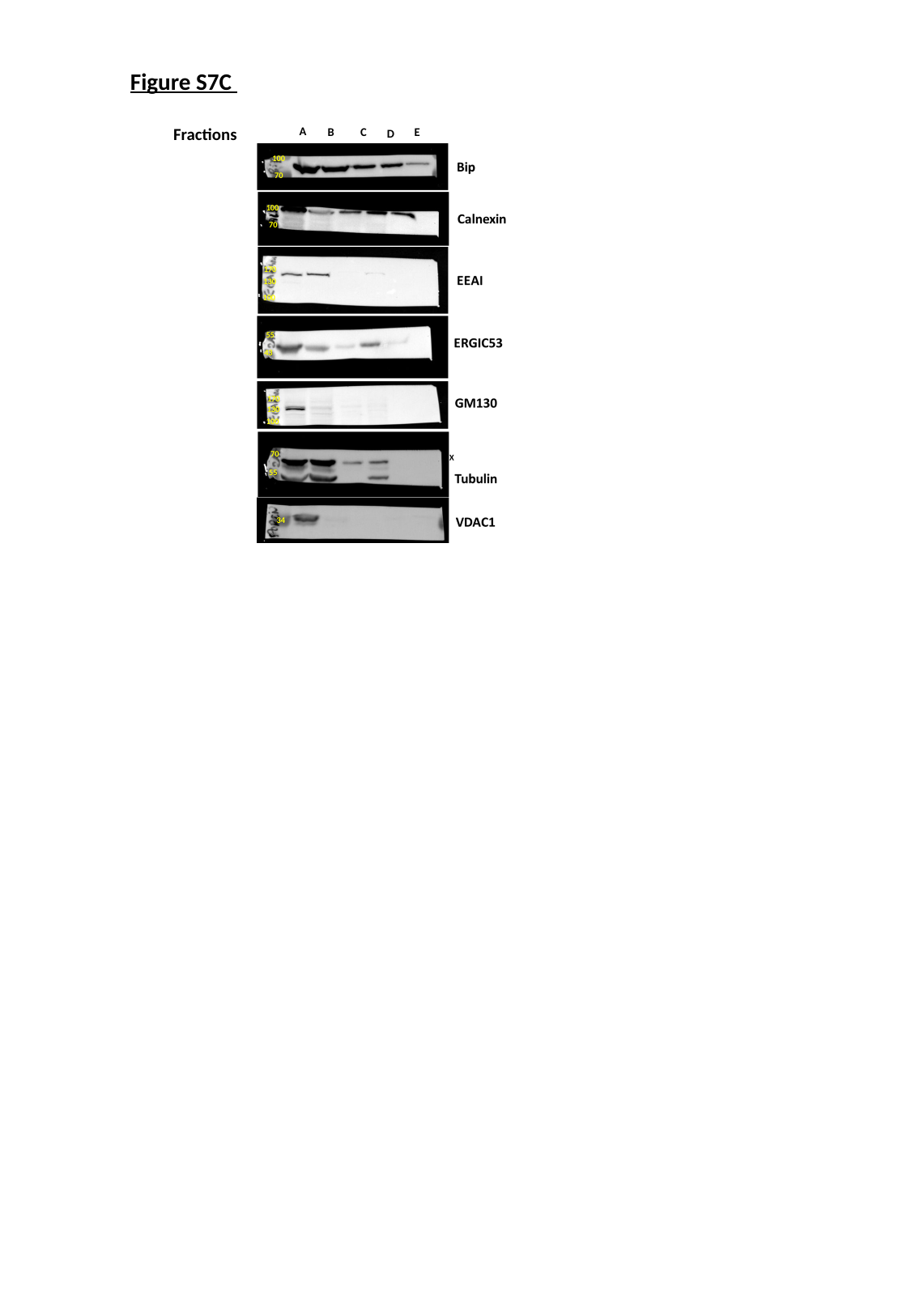

Figure S7C
Fractions
A
B
E
C
D
100
70
100
70
170
130
100
55
43
170
130
100
70
55
34
